# RASER MRI: Magnetic resonance images formed spontaneously exploiting cooperative nonlinear interaction

**DOI:** 10.1126/sciadv.abp8483

**Published:** 2022-07-13

**Authors:** Sören Lehmkuhl, Simon Fleischer, Lars Lohmann, Matthew S. Rosen, Eduard Y. Chekmenev, Alina Adams, Thomas Theis, Stephan Appelt

**Affiliations:** ^1^ Institute of Microstructure Technology, Karlsruhe Institute of Technology, 76344 Eggenstein-Leopoldshafen, Germany.; ^2^ Department of Chemistry, North Carolina State University, Raleigh, NC 27606, USA.; ^3^ Institute of Technical and Macromolecular Chemistry, RWTH Aachen University, 52056 Aachen, Germany.; ^4^ Massachusetts General Hospital, A. A. Martinos Center for Biomedical Imaging, Boston, MA 02129, USA.; ^5^ Department of Physics, Harvard University, Cambridge, MA 02138, USA.; ^6^ Department of Chemistry, Integrative Biosciences (IBio), Karmanos Cancer Institute (KCI), Wayne State University, Detroit, MI 48202, USA.; ^7^ Russian Academy of Sciences, Leninskiy Prospekt 14, Moscow 119991, Russia.; ^8^ Department of Physics, North Carolina State University, Raleigh, NC 27695, USA.; ^9^ Joint Department of Biomedical Engineering, University of North Carolina at Chapel Hill and North Carolina State University, Raleigh, NC 27695, USA.; ^10^ Central Institute for Engineering, Electronics and Analytics – Electronic Systems (ZEA-2), Forschungszentrum Jülich GmbH, D-52425 Jülich, Germany.

## Abstract

The spatial resolution of magnetic resonance imaging (MRI) is limited by the width of Lorentzian point spread functions associated with the transverse relaxation rate 1/*T*_2_^*^. Here, we show a different contrast mechanism in MRI by establishing RASER (radio-frequency amplification by stimulated emission of radiation) in imaged media. RASER imaging bursts emerge out of noise and without applying radio-frequency pulses when placing spins with sufficient population inversion in a weak magnetic field gradient. Small local differences in initial population inversion density can create stronger image contrast than conventional MRI. This different contrast mechanism is based on the cooperative nonlinear interaction between all slices. On the other hand, the cooperative nonlinear interaction gives rise to imaging artifacts, such as amplitude distortions and side lobes outside of the imaging domain. Contrast mechanism and artifacts are explored experimentally and predicted by simulations on the basis of a proposed RASER MRI theory.

## Teaser

Magnetic resonance images are generated without radio-frequency irradiation, relying on self-organization instead.

## INTRODUCTION

RASER [radio-frequency (RF) amplification by stimulated emission of radiation], also referred to as Zeeman maser, is a nuclear magnetic resonance (NMR) phenomenon as a result of stimulated nuclear spin transitions. RASERs have been investigated using hyperpolarized rare gases (*1*–*4*) as well as ^1^H, ^17^O, and even ^27^Al spins in liquids and solids (*5*–*9*). Multimode RASERs enable comagnetometry, which, in turn, allows for precision measurements (*10*–*13*). In addition, multimode RASER activity gives insight into fundamental phenomena in nonlinear mathematics (*14*) and synergetics (*15*) such as line collapse, multiple-period doubling, intermittence, and chaos (*4*, *12*, *16*). Most recently, the parahydrogen (*p*-H_2_) pumped (*17*, *18*) RASER has been established (*12*, *16*, *19*–*21*), by creating strong population inversions directly in room-temperature solutions. RASER magnetic resonance imaging (MRI) is associated with an alternative contrast mechanism than standard MRI, and it appears natural to wonder whether it could serve as a means to overcome fundamental limits of Lorentzian-based point spread functions (PSFs) in MRI (*22*, *23*).

The spatial resolution of MRI is limited by the width *w* = 1/(π*T*_2_*) of the Lorentzian PSF. Here, we show that nonlinearly coupled slices can spontaneously form an image out of nuclear spin noise, as an alternative to the superposition of uncoupled Lorentzian PSFs. We describe previously unknown nonlinear MRI physics in a *p*-H_2_ RASER while noting that nonlinear spin evolution in the presence of a gradient including radiation damping effects and dipolar fields has been reported before (*24*–*26*). We note that other hyperpolarization techniques may be used for RASER MRI as described here (*7*).

Conventional MRI uses spin or gradient echoes of nuclear magnetization that need to be excited with RF pulses. An interesting alternative is spin noise imaging, which measures projections without external RF excitation and fast gradient switching (*27*). Spin noise imaging does not require any initial hyperpolarization procedure but requires cryogenically cooled NMR probes and averaging to compensate for the low signal-to-noise ratio (SNR ~ 1).

The system under study here uses hyperpolarized samples in combination with an external high *Q* resonator at room temperature (*28*), thereby achieving an SNR of >200 in a single scan. The spontaneous RASER burst, which forms in the absence of external RF excitation, reflects the superposition of nonlinearly coupled slices. The corresponding spectrum (RASER MRI) of the burst reports on the spatial distribution of the samples spin number density and can have complicated and distorted shapes. On the other side, the image is very sensitive to local variations in the input profile. Therefore, RASER MRI entails new MRI physics challenges and opportunities caused by the nonlinear coupling.

In the presented work, RASERs emerge when placing a proton spin 1/2 ensemble with a large initial population inversion *d*_0_ = *N*_2_ − *N*_1_, above the RASER threshold $d_{\text{th}}=4V_{s}/(\mu_{0}\hbar \gamma_{H}^{2}\;T_{2}^{*}Q)$ in a resonant LC circuit with quality factor *Q*. In this system, *N*_2_ and *N*_1_ are the populations of the corresponding Zeeman levels 2 and 1; *V*_s_ is the sample volume; and μ_0_, ℏ, and γ_H_ denote the vacuum permeability, Planck’s constant, and the proton gyromagnetic ratio, respectively. For RASER MRI, the proton spins are first pumped into a state of highly negative spin polarization *P*_H_. This corresponds to a positive *d*_0_ value, which is assumed to be several orders of magnitude above the RASER threshold *d*_th_. An equivalent and convenient way to characterize the threshold condition for one singular mode is given by ε = *d*_0_/*d*_th_ = *T*_2_*/τ_rd_ ≫ 1 (*29*, *30*), where ε is a dimensionless quantity. Note that ε is the enhancement above the RASER threshold, not above thermal nuclear spin polarization. The radiation damping rate is given by $\tau_{\text{rd}}^{-1}=\mu_{0}\hbar \gamma_{H}^{2}\;Q\;d_{0}/(4V_{s})$, which includes inverted states (positive *d*_0_), and has been studied extensively in NMR spectroscopy (*24*, *29*, *31*–*33*).

To understand how the RASER can be used for MRI, we introduce an analysis of the RASER action in the presence of a magnetic field gradient *G_z_*. The gradient creates a frequency range Δ = γ_H_ · *G_z_* · *L* that spans the image domain of the object of length *L* (section S1). The initial nuclear spin population inversion is spread over the imaging domain Δ and is given by$\;d_{0}=\int_{\nu_{0}-\Delta /2}^{\nu_{0}+\Delta /2}\rho_{d}(\nu )d\nu$, where ρ_d_(ν) is the population inversion density and ν_0_ is the off-resonance frequency in the center of the imaging domain. The integrand ρ_d_(ν)*d*ν can be described as the number of negatively polarized spins in the frequency interval [ν, ν + *d*ν]. Given a profile ρ_d_(ν), a total RASER MRI signal emerges spontaneously out of nuclear spin noise.

To generate a system where a numerical evaluation is feasible, we divide the image domain Δ into *N* = Δ/δν individual slices. To avoid numerical artifacts, the distance δν between consecutive slices has to be chosen small enough. Specifically, δν < *w* has to be fulfilled, where *w* = 1/(π*T*_2_*) is the natural linewidth. Furthermore, to estimate whether a given *d*_0_ is RASER active in a given gradient *G_z_*, we also introduce the threshold population density ρ_d_^th^ = *d*_th_/*w* as used below.

To calculate the dynamics of the nonlinearly coupled slices, each slice μ = 1, …, *N* is characterized by an initial population inversion $d_{\mu }(0)=\int_{\nu_{0}-\Delta /2+(\mu -1)\delta \nu }^{\nu_{0}-\Delta /2+(\mu )\delta \nu }\rho_{d}(\nu )d\nu$. With a given initial *d*_μ_(0), the time evolution of the RASER modes or slices can be modeled by a set of μ = 1, …, *N* nonlinearly coupled differential equations for the population inversion *d*_μ_ and the transverse spin component α_μ_ = *A*_μ_ exp(*i*ϕ_μ_)

$${\overset{\cdot }{d}}_{\mu }=-\frac{d_{\mu }}{T_{1}}-4\beta \sum_{\sigma ,\tau =1}^{N}A_{\sigma }A_{\tau }\text{cos}(\phi_{\sigma }-\phi_{\tau })$$ (1)

$${\overset{\cdot }{A}}_{\mu }=-\frac{A_{\mu }}{T_{2}^{*}}+\beta d_{\mu }\sum_{\tau =1}^{N}A_{\tau }\text{cos}(\phi_{\tau }-\phi_{\mu })$$ (2)

$${\overset{\cdot }{\phi }}_{\mu }=2\pi \{\nu_{0}-0.5[\Delta -\delta \nu (2\mu -1)]\}+\beta \frac{d_{\mu }}{A_{\mu }}\sum_{\tau =1}^{N}A_{\tau }\text{sin}(\phi_{\tau }-\phi_{\mu })$$ (3)

$$d_{\mu }(0)=\int_{\nu_{0}-\Delta /2+(\mu -1)\delta \nu }^{\nu_{0}-\Delta /2+\mu \delta \nu }\rho_{d}(\nu )d\nu$$ (4)

The coupling constant β is given as $\beta =\mu_{0}\hbar \gamma_{H}^{2}Q/(4V_{s})$. The model for RASER MRI represented by Eqs. 1 to 4 is formulated in the rotating frame (for a complete derivation, see section S1) and is a modification of the existing multimode RASER theory (*12*, *16*). The modifications comprise the initial boundary conditions for *d*_μ_(0) in Eq. 4, the absence of pumping in Eq. 1, and the definition of the slice frequencies in Eq. 3. Numerical simulations of Eqs. 1 to 4 reveal three important invariance principles for RASER MRI: Provided that δν << *w* and *T*_1_ >> *T*_2_*, the shape of the RASER images is independent of (i) the value of the slicing δν, (ii) the longitudinal relaxation time *T*_1_, and (iii) the values of the initial conditions *A*_μ_(0) and ϕ_μ_(0) (see section S6). According to the invariance principle (iii), the shape of the RASER image is the same, irrespective that the initial conditions for *A*_μ_(0) and ϕ_μ_(0) are random values (i.e., nuclear spin noise) or a weak RF pulse with fixed values for *A*_μ_(0) and ϕ_μ_(0). The three invariance principles are crucial for RASER MRI, because they guaranty reproducibility of RASER MRI.

Certain processes can be identified by examining the dynamics described by Eqs. 1 to 3: The population inversion of a given mode μ in Eq. 1 decays with the rate 1/*T*_1_ and is decreased by the rate given by the sum over all quadratic terms −4β*A*_σ_*A*_τ_ cos (ϕ_σ_ − ϕ_τ_). In turn, the amplitude of *A*_μ_ in Eq. 2 decays with the rate 1/*T*_2_* and increases for τ = μ with the rate β*d*_μ_. The last term on the right side of Eq. 2, $\beta d_{\mu }\sum_{\tau =1}^{N}A_{\tau }\text{cos}(\phi_{\tau }-\phi_{\mu })$for τ ≠ μ, involves a sum over all other amplitudes *A*_τ_ cos (ϕ_τ_ − ϕ_μ_). This sum can be a growth or decay rate for *A*_μ_, depending on the specific values of all other phase differences ϕ_τ_ − ϕ_μ_. The collective action of all modes strongly influences the amplitude and sign of the rate *dA*_μ_/*dt*, which defines the amplitudes *A*_μ_ of the final image.

The spatial encoding of each slice μ = 1, …, *N* is reflected by the first term in Eq. 3, where each slice is oscillating at the angular frequency ω_μ_ = 2π(ν_0_ − 0.5 (Δ − δν(2μ − 1))). Apart from this linear evolution of ϕ_μ_ with time *t*, there is a nonlinear collective term $\left(\beta d_{\mu }/A_{\mu })\sum_{\tau =1}^{N}A_{\tau }\;\text{sin}(\phi_{\tau }-\phi_{\mu }\right)$, which is responsible for synchronism. Equation 3 is analogous to Kuramoto’s model of synchronized oscillators (*34*–*36*). The dynamics of RASER MRI given by Eqs. 1 to 4 can be described by a collection of synchronized oscillators or slices with distinct angular frequencies ω_μ_, where the amplitude *A*_μ_ of each oscillator depends on the self-organization controlled by the collective interaction with all other slices. Therefore, the derivative of the amplitude of each slice depends on the mean-field amplitude produced by all other slices.

**Fig. 1.**
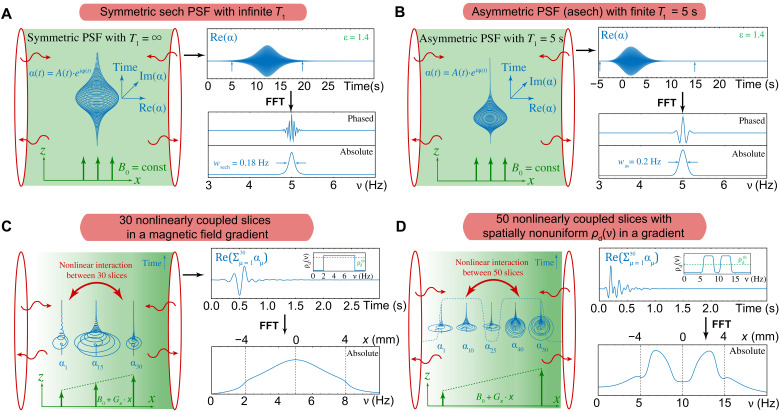
Simulated RASER signals and the corresponding Fourier-transformed spectra for different numbers of interacting slices. The nonlinear interaction between all slices is mediated by the virtual photons (wavy arrows, wavelength ≫ sample dimension) in the resonator (red) (*51*). After the RASER burst, the Zeeman energy of the spins is fully transferred to the current of the coil (*52*). (**A**) For *N* = 1 and *T*_1_ = ∞, the signal α_1_(*t*) = α(*t*) is plotted in the [*t*, Re(α), Im(α)] space (left). The projection Re(α) for *d*_0_ = 4.2 · 10^15^ and the corresponding Fourier-transformed spectra are shown on the right. (**B**) For *N* = 1, *T*_1_ = 5 s and *d*_0_ = 4.2 · 10^15^, the signal burst α(*t*) is asymmetric with respect to time. (**C**) Sketch of three representative signals α_μ_, where μ = 1, 15, and 30 of *N* = 30 interacting slices [*T*_1_ = 5 s, Δ = 6 Hz, rectangular profile with ρ_d_(ν) = 7.5 · 10^15^/Hz]. (**D**) Five representative signals α_μ_ of *N* = 50 coupled slices [*T*_1_ = 5 s, Δ = 10 Hz, nonuniform density ρ_d_(ν)]. Threshold population density ρ_d_^th^ = *d*_th_/*w* = 6.6 · 10^15^/Hz is indicated as dotted line in the insets in (C) and (D). FFT, fast Fourier transform.

Last, the total RASER signal is obtained by the sum of all transverse spin components $\text{Sig}(t)=N^{-1/2}\sum_{\mu =1}^{N}\text{Re}(\alpha_{\mu })=N^{-1/2}\sum_{\mu =1}^{N}A_{\mu }\text{Re}(\text{exp}[i\phi_{\mu }])$, where *N*^−1/2^ is a normalization constant. Here, we focus on the difference between the concept of single PSFs to analyze conventional magnetic resonance image formation and the collective mean-field approach, which is the basis of RASER MRI. Numerical solutions of Eqs. 1 to 4 are evaluated (see Fig. 1) to highlight the difference of the spin dynamics for a single RASER slice and the collective behavior of coupled slices.

The simplest case is shown in Fig. 1A for *N* = 1 and *T*_1_ = ∞, where the numerically evaluated form matches the exact solution introduced by Mao *et al.* (*31*, *37*, *38*) and discussed by others (*39*, *40*). The corresponding phased and absolute spectra of α *=* α_1_ are displayed Fig. 1A (bottom right). For this case, *T*_1_ = ∞, the PSF is a hyperbolic secant with width *w*_sech_ (section S2 and eq. S19). Close to the threshold, such a PSF is narrower than the Lorentzian NMR linewidth *w* = 1/(π*T*_2_*), because the RASER signal involves dedamping.

No exact solution exists for a finite *T*_1_, but the MR signal represents an asymmetrically shaped PSF (Fig. 1B and section S3). The linewidth *w*_as_ in the spectrum is slightly broader compared to the symmetric case (Fig. 1A) but still smaller than *w*.

Here, we include both the effects of finite *T*_1_ and the nonlinear interactions between *N* slices formed in the presence of a gradient. In contrast to standard MRI, the image contrast and the spatial resolution cannot be explained by independent individual PSFs. Each slice is sensitive to the collective action of all slices, which makes RASER imaging highly sensitive to local variations in *d*_μ_ (section S4A), providing interesting avenues for future investigations for RASER MRI.

## RESULTS

### RASER MRI explored by numerical simulation

In the simulation in Fig. 1C, a rectangular polarization profile (inset, top right) is assumed to generate a RASER signal in the presence of a field gradient. The time evolution of three of the *N* = 30 slices is depicted on the left. The shape of signal of these slices differs significantly from the uncoupled PSFs in Fig. 1 (A and B). A corresponding one-dimensional (1D) RASER image (projection) is obtained as the Fourier transform from Sig(*t*) = Re(∑α_μ_). The amplitude in the center of the RASER image is larger, and decaying side lobes arise outside of the image boundaries at *x* = ±4 mm (bottom right). These artifacts are expected from the theory described in Eqs. 1 to 4 and evaluated in detail by numerical simulations in section S4.

**Fig. 2.**
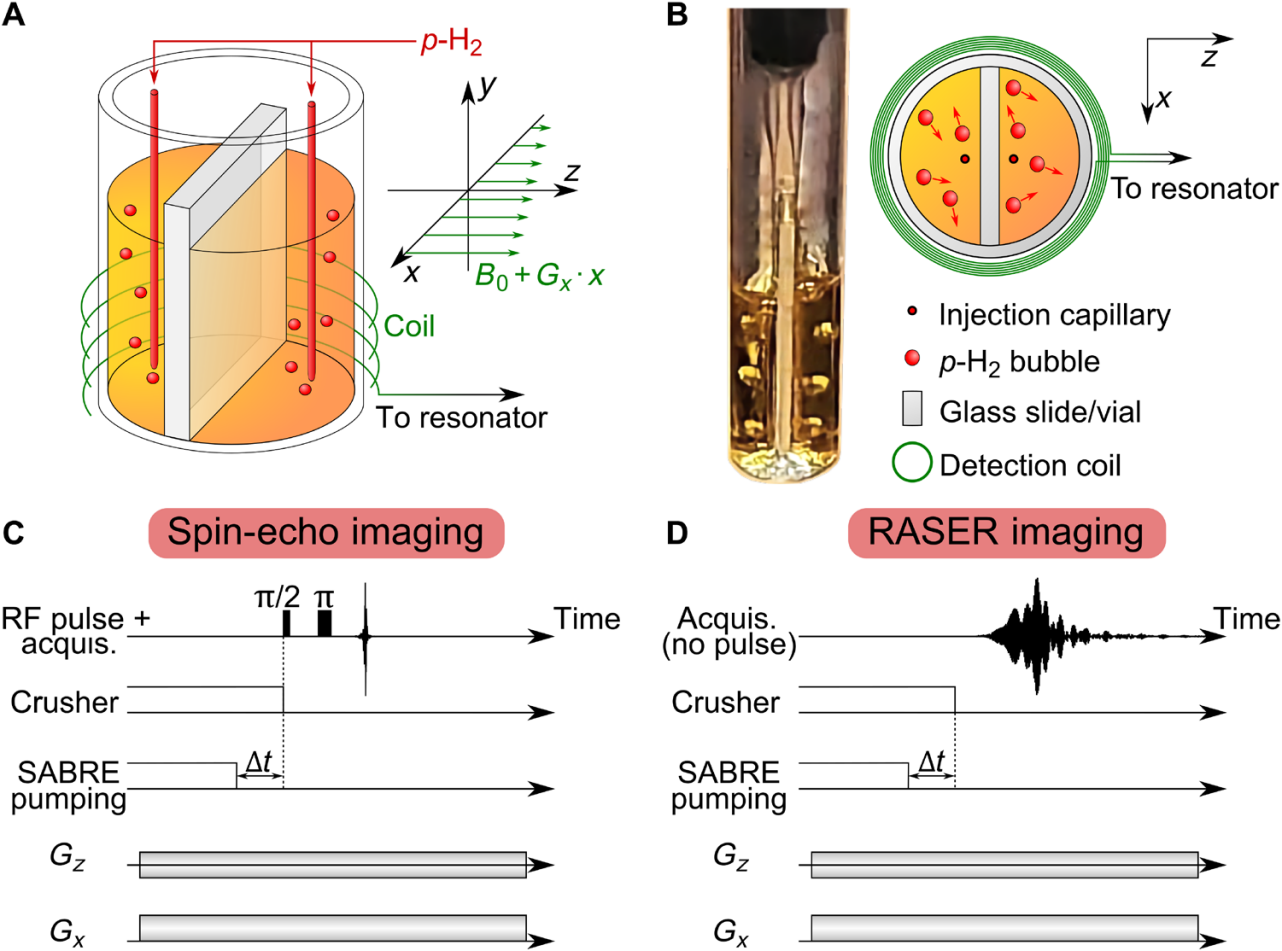
Experimental setup for MRI of a two-chamber phantom and corresponding pulse sequences for SEI and RASER MRI. (**A**) Schematics of the two imaged chambers and of the gradient directions. (**B**) Photo and top-down schematic of the two chambers (*L* = 8 mm in diameter and 10 mm in height separated by a 1-mm-thick glass slide) including bubbling of *p*-H_2_ through two capillaries [100 μm in outer diameter (OD) and 30 μm in inner diameter (ID)]. (**C**) Spin echo sequence of 90° followed by a 180° rf pulse for SEI. (**D**) RASER imaging sequence. For both imaging sequences, a crusher gradient is applied to destroy all coherence, while negative proton polarization is built up by SABRE pumping at magnetic fields *B*_0_ of 3.9 and 7.8 mT. *p*-H_2_ bubbling is interrupted to allow the solution to settle for a time Δ*t*. For SEI, the image is encoded in the echo signal. In the case of RASER MRI, the signal builds up spontaneously in the absence of any RF excitation. Frequency encoding is performed in the *x* and *z* directions.

In Fig. 1D, we simulate a RASER image using a spin density profile ρ_d_(ν) to match the experimental setup described in Fig. 2 (A and B). This nonuniform spin density profile ρ_d_(ν) entails two equal compartments separated by a gap. The evolution of five representative RASER slices of *N* = 50 coupled slices is shown (Fig. 1D, left). The image after Fourier transformation (bottom right) reflects roughly the shape ρ_d_(ν) except for the deformed amplitudes of the flat tops and the side lobes, which occur outside the imaging boundaries.

### Experimental realization of RASER MRI: 1D demonstrations

To experimentally examine the RASER MRI theory, a simple phantom was prepared consisting of a cylindrical sample chamber divided into two measurement chambers by a glass slide (Fig. 2, A and B). The two chambers are individually supplied with *p*-H_2_ to generate highly negative polarized proton spins (i.e., *d*_0_ ≫ *d*_th_). The chemical system chosen is pyrazine in a liquid methanol-*d*_4_ solution with a dissolved iridium-based Signal Amplification By Reversible Exchange (SABRE) catalyst for nuclear spin polarization (*18*, *41*). RASER magnetic resonance image were acquired in the presence of weak *G_x_* and *G_z_* magnetic field gradients on the order of a few milligauss per centimeter.

Conventional magnetic resonance images were obtained with a spin echo sequence of 90° followed by a 180° rf pulse (Fig. 2C) as a reference. Before the acquisition of the reference spin echo image (SEI), a crusher field gradient was applied to the hyperpolarized sample, to suppress spontaneous RASER buildup. 1D images were acquired using the *G_z_* gradient to visualize the two chambers separated by the dividing glass slide. 2D images were recorded through stepwise switching of the *G_x_* and *G_z_* gradients to rotate through a circle with constant absolute gradient [|*G*| =(*G_z_*^2^ *+ G_x_*^2^)^1/2^]. The 2D image was then obtained via projection reconstruction, which is also common in computed tomography.

The RASER images were acquired in a similar way (Fig. 2D), but in contrast to the spin echo sequence, no RF pulses were applied. The signal is acquired in the presence of *G_x_* and *G_z_* field gradients during spontaneous RASER emission, which begins shortly after the crusher field gradient is turned off.

**Fig. 3.**
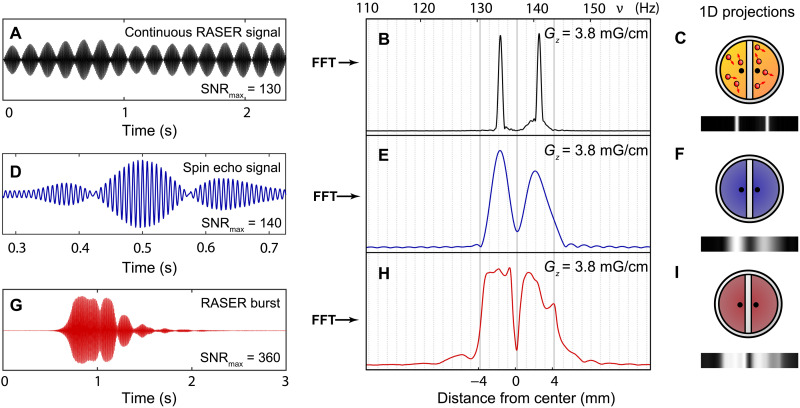
1D projections of a continuously pumped proton RASER, a SEI, and a RASER image. (**A**) Continuously SABRE pumped proton RASER signal and corresponding fast Fourier transform spectrum (**B**) in the presence of a gradient *G_z_*. A Hamming window is applied to the signal before fast Fourier transform to suppress sinc wiggles. (**D**) Spin echo acquired with the sequence in Fig. 2C and (**E**) corresponding Fourier-transformed SEI. (**G**) RASER burst acquired with the sequence in Fig. 2D. (**H**) Corresponding RASER 1D projection, which is three times better resolved (δ*z*_RI_ ≈ 90 μm) than the SEI in (E). *B*_0_ = 7.8 mT (proton resonance frequency of 333 kHz), and no slice selection is applied. The RASER image (H) has SNR_max_ = 360 at Δ*t* = 2 s, while the SEI in (B) yields SNR_max_ = 140 at Δ*t* = 5 s. All images are phased in the absolute mode and were measured in a single scan. (**C**, **F**, and **I**) Corresponding image phantom and 1D projections.

The RASER action can be measured over an indefinite period (Fig. 3A), when *p*-H_2_ is continuously bubbled through the solution. However, the bubbling-induced sample motion in the presence of field gradients is a challenge for imaging. The motion collapses the RASER spectrum in each chamber into one average frequency (Fig. 3B). To avoid line collapse induced by sample motion and to enable imaging, the *p*-H_2_ flow had to be stopped and an additional waiting time Δ*t* was introduced, which allows for the solution to settle and the motions to halt. Now, both spin echo and 1D RASER signals could be acquired (Fig. 3, D and G) shortly after the crusher gradient was switched off. The acquired RASER burst in Fig. 3G is significantly longer than the corresponding spin echo in Fig. 3D acquired at the same gradient strength of *G*_*z*_ = 3.84 mG/cm.

The spatial resolution limit is given by δ*z* = *w*/(γ_H_ · *G_z_*) in conventional MRI (*22*). This limit yields δ*z*_SEI_ = 280 μm for the SEI in Fig. 3E, and as a result, the gap and the edges of the sample are not well resolved. However, for RASER 1D projection in Fig. 3H, the slope at the image boundaries at the gap is more than three times steeper. This corresponds to an estimated spatial resolution of δ*z*_RI_ ≈ 90 μm. However, care has to be taken with this comparison because the contrast mechanism for RASER MRI is based on collective and nonlinear interaction. Spatial resolution might not be a suitable measure for the observed hole in Fig. 3H. Instead, we examine the sensitivity of RASER MRI to local variation in the object ρ_d_(ν) compared to the sensitivity of conventional MRI to local variations in the object. Simulations support that RASER MRI is more sensitive to small local variations in the imaged object (section S4A and fig. S7).

The measured 1D RASER image in Fig. 3H shows signal lobes outside the boundaries of *z* = −4 mm, in accordance with the simulation shown in Fig. 1D. These artifacts from 1D RASER MRI are analyzed in section S4B, and a potential correction method is proposed.

### Experimental realization of RASER MRI: 2D demonstration and comparison to traditional SEI of hyperpolarized solutions

**Fig. 4.**
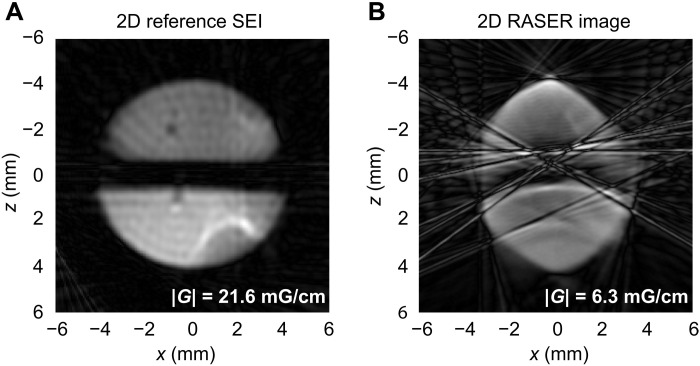
2D SEI and 2D RASER image. (**A**) 2D SEI and (**B**) 2D RASER MRI measured at 3.9 mT. The 2D images (A and B) are obtained by projection reconstruction of 30 projections each. These 1D projections are measured with the sequence in Fig. 2 (C and D) from different angles by varying *G_x_* and *G_z_* such that *G_x_*^2^ + *G_z_*^2^ = constant. In (A), the two capillaries used for *p*-H_2_ supply are visible around *x* = −1 mm, *z* = 0.5 mm and *x* = −1.5 mm, *z* = −2 mm for each chamber. The RASER image (B) is recorded at a 3.5 times smaller gradient than (A), but both spatial resolutions are similar. The RASER image is affected by interference lines. The origin of these artifacts is discussed in the text and in section S5.

Both a 2D SEI (Fig. 4A) and a 2D RASER MRI (Fig. 4B) of the same sample are obtained, extending 1D imaging to 2D imaging by reconstructing from 30 angular directions. The field gradient used for the SEI was 3.5 times larger than that for RASER MRI to obtain comparable resolution. Each individual projection in the SEI has a resolution of 50 μm, only about an order of magnitude higher than modern microimaging (*42*–*44*). The two semicircle-shaped halves and the 1-mm gap are visible in Fig. 4 (A and B). These images also display typical projection reconstruction star artifacts outside of the imaging domain. The 2D RASER image in Fig. 4B not only shows sharper features but also exhibits a deformed shape of the sample and its gap, paired with several interfering lines. These lines could be caused by the nonlinear interaction between the slices, analogous to features observed during strong radiation damping at high magnetic field (*45*). An alternative reason could be the residual motion in the liquid after turning off the *p-*H_2_ pumping. These artifacts can be identified in the individual 1D projections, which are used to reconstruct the 2D RASER image (see fig. S11).

### RASER MRI dependence on polarization

**Fig. 5.**
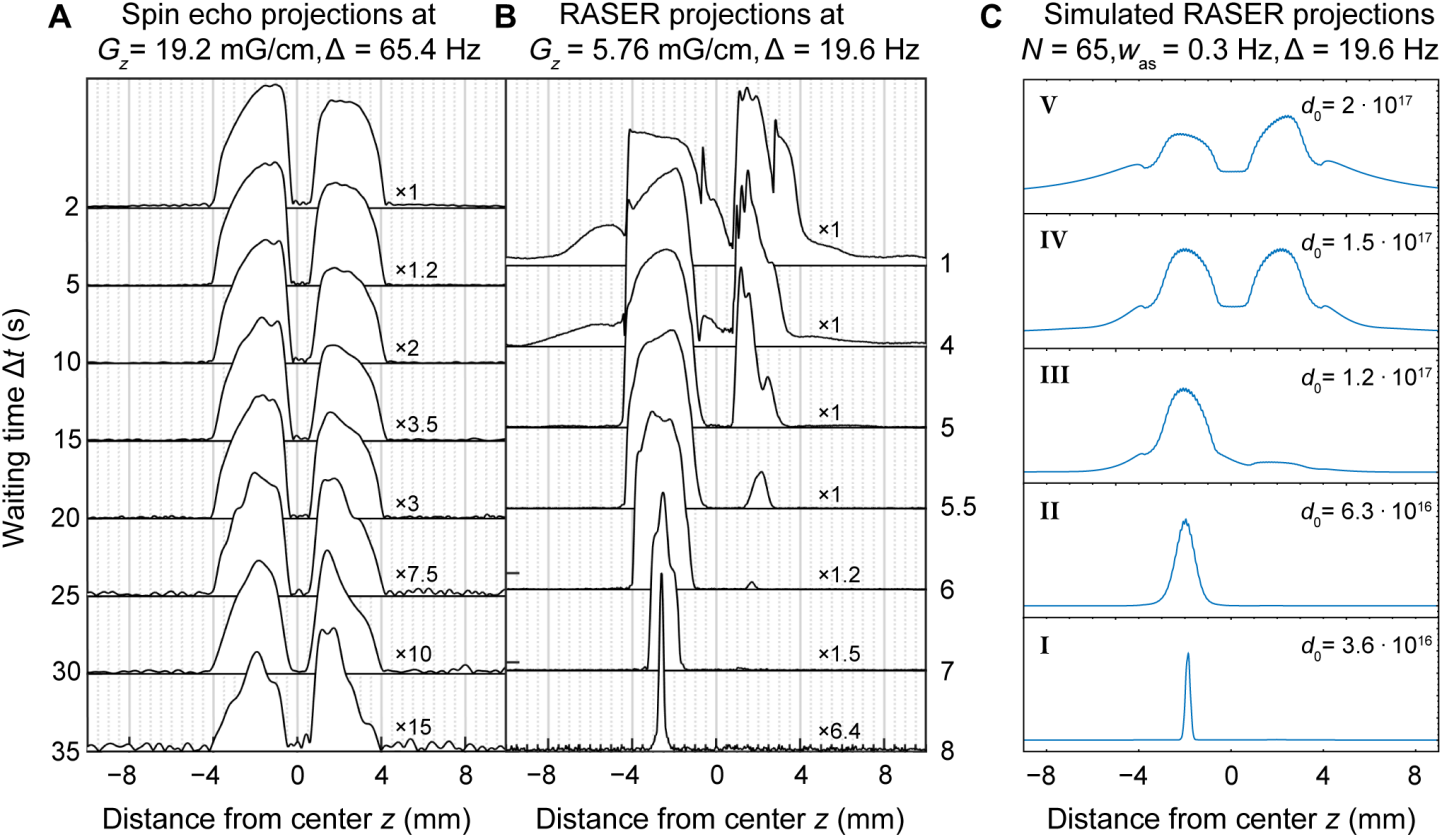
Projections of measured SEI, RASER MRI at *B*_0_ = 7.8 mT, and simulated RASER MRI at different waiting times Δ*t*. (**A**) The SEI was acquired at *G_z_* = 19.2 mG/cm (δ*z*_SEI_ = 0.055 mm) without slice selection. The shape remains form invariant up until Δ*t* = 20 s. (**B**) The 1D RASER image was acquired at *G_z_* = 5.76 mG/cm. At higher polarizations, i.e., for Δ*t* < 5 s, both sides of the image are governed by strong nonlinear effects. At lower polarization, Δ*t* > 5 s, the amplitude of the right half in the phantom is strongly attenuated. At Δ*t* = 8 s, the RASER image is reduced to one peak of 0.6 Hz width. (**C**) Simulated RASER images, based on Eqs. 1 to 4 and on a profile ρ_d_(ν) similar to the SEI in (A). These reflect the basic features at different values *d*_0_ (I to V), i.e., side lobes outside of the imaging domain and nonlinear deformations. All spectra are phased in absolute mode and normalized to the maxima of each image.

A stark contrast of RASER MRI to traditional MRI is the dependence of RASER MRI images on the magnitude of the nuclear spin polarization. Figure 5 shows a series of 1D RASER images and SEIs of the phantom, acquired with decreasing levels of polarization, i.e., decreasing population inversion *d*_0_. The polarization was adjusted by implementing an increasing waiting time Δ*t* between the polarization step and acquisition.

For SEI, decreasing polarization entails decreasing SNR for each image in Fig. 5A, but the shape of the image in the interval of 2 s < Δ*t* < 20 s (about a few *T*_1_ relaxation periods) remains invariant. The spatial resolution for the SEI is determined by the slope on the sample boundaries with δ*z*_SEI_ ≈ 50 μm. This observation is in overall good agreement with the theoretical expectation of δ*z*_SEI_ = *w*/(γ_H_ · *G_z_*) = 55 μm. Although the initial negative polarization (*d*_0_) changes by more than a factor 10 within the first 20 s, the shape of the SEIs is invariant. This behavior exists because the widths of the underlying PSFs barely deviate from a Lorentzian linewidth and radiation damping effects are insignificant. At longer waiting times (Δ*t* > 20 s), noise becomes more dominant, and the shape deteriorates as more efficient relaxation at the walls decreases the image amplitude at the boundaries of the sample.

In contrast, the RASER image shape in Fig. 5B strongly depends on polarization. We attribute the differences between the two image halves to disparities in the bubbling rates and phantom shapes (see section S4C). In the case of low polarization (Δ*t* > 15 s), the maximum amplitude of the right half of the sample is substantially smaller, because the population inversion density ρ_d_(ν) is closer to the RASER threshold density ρ_d_^th^. Because of the collective and nonlinear nature of the contrast mechanism, the slightly larger ρ_d_(ν) of the left half substantially suppresses the amplitude on the right half. This asymmetry in amplitude is much less pronounced if ρ_d_(ν) is further above ρ_d_^th^, for example, at a weaker gradient *G_z_* as in Fig. 3H. Figure 5C shows simulated RASER images for five different initial population inversions *d*_0_ and corresponding profiles ρ_d_(ν) (see fig. S10) to examine the origin of the RASER image distortions. The experiment at Δ*t* = 8 s matches the simulation with only one peak (width = 0.6 Hz; fig. S9), and for the experiments Δ*t* < 8 s, the simulation qualitatively reflects the amplitude deformations and side lobes seen in the measured images. The ripples in some images in Fig. 5B cannot be simulated assuming a uniform division of the RASER image into *N* = Δ/δν slices. Motional artifacts and variations of *T*_1_, *T*_2_^*^, and *B*_1_ field over the image domain may be responsible for the observed ripples.

## DISCUSSION

The proof-of-principle experiments provided here and the corresponding nascent theoretical framework motivate several new challenges and may provide an opportunity to explore the power of alternative contrast mechanisms provided by RASER MRI. A high sensitivity with respect to local variations in the input profile was found, which is based on collective nonlinear interactions between all regions of the sample. There is negligible background signal from other protons (e.g., water or solvent) in RASER experiments. At low magnetic fields (4 and 8 mT are demonstrated here), the RASER signal is many orders of magnitude larger compared to the signal of the more abundant background protons with low Boltzmann polarization. At higher magnetic fields (1.4 T) in RASER NMR spectroscopy, no proton background signals of water were observed because the RF from the RASER active protons does not excite the chemically shifted water protons (*46*). Further potential advantages are the absence of external RF excitation (*27*) (e.g., caused by the transmission coil), which imply minimal specific absorption rate, avoiding unintended heat deposition. In addition, RASER MRI can produce sufficient contrast with weaker magnetic field gradients, reducing potential concerns over peripheral nerve stimulation (*47*). This is a relevant concern if in vivo translation is possible. Last, the RASER MRI theory is connected to many seemingly disjunct systems in science and technology. The developed system of differential equation (Eqs. 1 to 4) and its solutions for the RASER MRI model are equivalent to the fundamental equations in many other fields with prominent examples in synergetics (*15*) and nonlinear dynamics (*14*, *36*, *48*, *49*). We point the interested reader to section S6, where several of those analogies are detailed.

## MATERIALS AND METHODS

### Sample preparation and setup

SABRE samples were prepared under Schlenk conditions. The samples contained 5 mM SABRE catalyst precursor [Ir(cyclooctadiene)(1,3-bis(2,4,6-trimethylphenyl)imidazole-2-ylidene)Cl] (*41*), and *c*_pyr_ = 100 mM pyrazine in methanol-*d*_4_. Pyrazine was chosen because it is associated with a single resonance in the NMR spectrum with *n*_s(pyr)_ = 4 chemically and magnetically equivalent protons, ideal for RASER and SEI experiments. Three hundred microliters were filled into each chamber, giving a total sample volume *V*_s_ = 600 μl. A glass capillary [~100 μm in outer diameter (OD) and 30 μm in inner diameter (ID)] was introduced into each chamber for parallel *p*-H_2_ supply. During polarization buildup, *p*-H_2_ was bubbled through the solution at a flow rate of ~30 sccm and at pressure of 2 bar. *p*-H_2_ was generated using a Bruker *p*-H_2_ generator at 35 K, yielding ~94% enriched *p*-H_2_ gas. The sample is located in a cylindrical glass tube (ID = 8 mm), divided by a glass slide (1 mm in thickness) for two-chamber experiments. The designed phantom is handmade. The 1-mm-thick glass sheet is held in place by chemically resistant glue. The liquid sample inside the two chambers is located in the sensitive volume of a cylindrical NMR detection coil (10 mm in ID and 10 mm in height), which is connected to an external resonator with high quality factor (*Q*_ext_ = 360 at 166 kHz) for sensitive detection of the NMR or RASER signals (*28*). Typically, a negative pyrazine proton polarization of *P*_H_ ≈ −10^−3^ to −10^−2^ is achieved in a magnetic field ranging from 3.9 to 7.8 mT. These chosen magnetic fields are close to the field *B*_0_ = 6.5 mT, where the SABRE ^1^H polarization for pyridine and similar chemical motives such as pyrazine is maximized (*18*). With respect to RASER MRI, low magnetic fields do offer the additional advantage of lower susceptibility artifacts.

### Setup-specific parameters

A SABRE-induced ^1^H polarization of *P*_H_ = −10^−3^ corresponds to a population inversion *d*_0_ = *c*_pyr_ · *V*_s_ · (−*P*_H_) · *n*_s(pyr)_ *N*_A_ = 0.1 mol/l · 6 · 10^−4^ l · −(−10^−3^) · 4, 6.022·10^23^/mol = 1.4 · 10^17^. The total number of ^1^H spins in the sample is *N*_s_ = 1.4 · 10^20^. Analogous calculations yield the initial conditions for simulations in RASER MRI explored by numerical simulation and RASER MRI dependence on polarization and the Supplementary Materials. For example, in Fig. 5, the initial population inversion is assumed to lie between *d*_0_ = 3.6 · 10^16^ and 2 · 10^17^. The ^1^H NMR parameters of pyrazine were measured to be *T*_2_* = 0.7 s (Lorentzian width *w* = 1/(π*T*_2_*) = 0.455 Hz). *T*_1_ values at different positions were measured using the results of the SEIs versus Δ*t* (see Fig. 5A). We found *T*_1_ = 5.0 s in the bulk. The measurement close to the walls varied around *T*_1_ = 2.5 ± 0.5 s. For the simulations, we chose a difference in *T*_1_ between the bulk and the walls of 3 s.

The total quality factor of the combined resonator (external resonator and NMR coil) is *Q* = 100. The *B*_1_ field profile from the NMR detection coil in the center of the sample is calculated to be about 10% lower compared to the field at the edges of the sample. As the RASER active slices interact through the *B*_1_ field of the coil, the coupling now depends on space, which is not accounted for in the parameter β in Eqs. 1 to 3. In summary, the dependence of *B*_1_, *T*_2_*, and *T*_1_ on the location of the sample is the major sources for RASER imaging artifacts. Correction algorithms for artifacts are state of the art for high-field MRI scanners (*50*) and could mostly be adapted to the artifacts presented here. The magnetic fields of the low-cost MRI system are generated by a set of four handmade shim gradients (*G_x_*, *G_y_*, *G_z_*, and *G*_crush_) and an electromagnet producing a constant field in the range of 0.5 mT < *B*_0_ < 20 mT. For our experiments, we chose *B*_0_ = 3.9 and 7.8 mT corresponding to 166.6- and 333.3-kHz ^1^H resonance frequency, respectively. The reference frequency of the spectrometer is chosen such that the off-resonance frequency ν_0_ is between 20 and 150 Hz away from the ^1^H resonance frequency. The homogeneity of the *B*_0_ field is 1 part per million (ppm)/cm^3^. The *p*-H_2_ supply in a low-field electromagnet in conjunction with sensitive external high-quality-factor enhanced (EHQE) detection avoids the necessity of a shuttling system for rapid transport of the sample into a high-field magnet. The *G_x_* and *G_z_* gradients were used to obtain projections from 30 different angles (in 6° steps). All data were acquired in a single scan. SEIs were acquired at an echo time of 1 s. 2D images were obtained after projection reconstruction of the 1D slices using a MATLAB code, written for this project. The spatial resolution is divided into a resolution along a slice in radial and angular direction. The radial resolution is 50 μm for SEI at 21.6 mG/cm, which corresponds to 160 points along the 8-mm sample diameter. The angular resolution with 30 slices spanning 180° is 6°.

There are frequency shifts due to slow magnetic *B*_0_ field drifts in the order of a few ppm per minute. At 333 kHz (7.8 mT), these drifts on a time scale of 10 min were more pronounced compared to 166 kHz (3.9 mT). The reason is thermal instability of the current supply in conjunction with heating of the resistive *B*_0_ field coil. For one 1D RASER image measured at 7.8 mT with a corresponding RASER burst lasting a few seconds, a drift of a ppm per minute means less than 0.1 ppm or 0.03-Hz frequency drift. The image domain Δ is typically chosen between 10 and 100 Hz (corresponding to about 20 to 200 slices for SEI), so the drift for a single 1D RASER image is negligible. For a 2D RASER image with a total measuring time of about 30 min for all 30 1D slices, the central frequency between the individual 1D slices could differ by a few Hz. Thus, each 1D image was shifted to yield the same center frequency for all 1D images before projection reconstruction.

### Simulation details

The simulations based on the model Eqs. 1 to 4 were performed using Mathematica 8. The NDSolve[] routine was used for the numerical evaluation of the variables *d*_μ_(*t*), *A*_μ_(*t*), and ϕ_μ_(*t*). The computation time of the system eqs. S5 to S8 can be quite long depending on the number of modes *N*. All parameters *d*_μ_, *A*_μ_, and ϕ_μ_ are coupled in between each other in a nonlinear way by the cos and sin terms on the right sides of eqs. S5 to S7. This is the reason for many nonlinear phenomena, which can arise in this RASER MRI model, ranging from phase locking, collapse phenomena, nonlinear image distortions, and edge effects to multiple-period doubling and chaos. While there are exactly *N* coupling terms for *A*_μ_ and ϕ_μ_ in eqs. S6 and S7, the number of coupling terms for *d*_μ_ in Eq. S5 is *N*(*N* − 1)/2. For larger numbers of slices, *N* > 100, the system of equations becomes elaborate and a large amount of computation is required. The computation time is roughly proportional to *N*^3^, so the system eqs. S5 to S7 is classified as a polynomial problem. A typical numerical evaluation using a personal computer takes about 60 s for *N* = 50 and can be many hours to days for *N* > 100.

For these simulations, initial conditions for *d*_μ_(0), *A*_μ_(0), and ϕ_μ_(0) are required. The initial conditions for *d*_μ_(0) at *t* = 0 were calculated for a given profile ρ_d_(ν) (Eq. 4). For *N*_s_ = 1.4 · 10^20 1^H spins, the average value for the initial spin noise amplitude is <*A*> ~ (*N*_s_)^1/2^ = 1.18 · 10^10^ with a random phase ϕ_μ_(0). For the simulations, constant values were assumed for simplicity [i.e., *A*_μ_(0) = 10^12^ and ϕ_μ_(0) = 0] because the RASER image is independent from the initial transverse spin components [see invariance principle (III) in Introduction and section S1).

## Supplementary Materials

### This PDF file includes:

Supplementary Text Figs. S1 to S12 References

## References

[1] M. G. Richards, B. P. Cowan, M. F. Secca, K. Machin, The ^3^He nuclear Zeeman maser. J. Phys. B. At. Mol. Opt. 21, 665–681 (1988).

[2] T. E. Chupp, R. J. Hoare, R. L. Walsworth, B. Wu, Spin-exchange-pumped ^3^He and ^129^Xe Zeeman masers. Phys. Rev. Lett. 72, 2363–2366 (1994). 10055861 10.1103/PhysRevLett.72.2363

[3] H. Gilles, Y. Monfort, J. Hamel, ^3^He maser for earth magnetic field measurement. Rev. Sci. Instrum. 74, 4515–4520 (2003).

[4] D. J. Marion, G. Huber, P. Berthault, H. Desvaux, Observation of noise-triggered chaotic emissions in an NMR-maser. Chem Phys. Chem. 9, 1395–1401 (2008). 18523949 10.1002/cphc.200800113

[5] P. Bösiger, E. Brun, D. Meier, Solid-state nuclear spin-flip maser pumped by dynamic nuclear polarization. Phys. Rev. Lett. 38, 602–605 (1977).

[6] A. G. Zhuravrev, V. L. Berdinskiǐ, A. L. Buchachenko, Generation of high-frequency current by the products of a photochemical reaction. JETP Lett. 28, 140 (1978).

[7] H. Y. Chen, Y. Lee, S. Bowen, C. Hilty, Spontaneous emission of NMR signals in hyperpolarized proton spin systems. J. Magn. Reson. 208, 204–209 (2011). 21145766 10.1016/j.jmr.2010.11.002

[8] E. M. M. Weber, D. Kurzbach, D. Abergel, A DNP-hyperpolarized solid-state water NMR MASER: Observation and qualitative analysis. Phys. Chem. Chem. Phys. 21, 21278–21286 (2019). 31549135 10.1039/c9cp03334c

[9] M. A. Hope, S. Björgvinsdóttir, C. P. Grey, L. Emsley, A magic angle spinning activated ^17^O DNP raser. J. Phys. Chem. Lett. 12, 345–349 (2021). 33355469 10.1021/acs.jpclett.0c03457

[10] S. Appelt, G. Wäckerle, M. Mehring, Deviation from Berry’s adiabatic geometric phase in a ^131^Xe nuclear gyroscope. Phys. Rev. Lett. 72, 3921–3924 (1994). 10056334 10.1103/PhysRevLett.72.3921

[11] T. W. Kornack, R. K. Ghosh, M. V. Romalis, Nuclear spin gyroscope based on an atomic comagnetometer. Phys. Rev. Lett. 95, 230801 (2005). 16384290 10.1103/PhysRevLett.95.230801

[12] S. Appelt, A. Kentner, S. Lehmkuhl, B. Blümich, From LASER physics to the para-hydrogen pumped RASER. Prog. Nucl. Magn. Reson. Spectrosc. 114–115, 1–32 (2019). 10.1016/j.pnmrs.2019.05.003 31779878

[13] V. V. Soshenko, S. V. Bolshedvorskii, O. Rubinas, V. N. Sorokin, A. N. Smolyaninov, V. V. Vorobyov, A. V. Akimov, Nuclear spin gyroscope based on the nitrogen vacancy center in diamond. Phys. Rev. Lett. 126, 197702 (2021). 34047600 10.1103/PhysRevLett.126.197702

[14] S. H. Strogatz, *Nonlinear Dynamics and Chaos: With Applications to Physics*, *Biology*, *Chemistry*, *and Engineering* (Avalon Publishing, 2014).

[15] H. Haken, *Synergertics: An Introduction* (Springer-Verlag, 1983).

[16] S. Appelt, S. Lehmkuhl, S. Fleischer, B. Joalland, N. M. Ariyasingha, E. Y. Chekmenev, T. Theis, SABRE and PHIP pumped RASER and the route to chaos. J. Magn. Reson. 322, 106815 (2021). 33423756 10.1016/j.jmr.2020.106815 PMC8026265

[17] C. R. Bowers, D. P. Weitekamp, Transformation of symmetrization order to nuclear-spin magnetization by chemical reaction and nuclear magnetic resonance. Phys. Rev. Lett. 57, 2645–2648 (1986). 10033824 10.1103/PhysRevLett.57.2645

[18] R. W. Adams, J. A. Aguilar, K. D. Atkinson, M. J. Cowley, P. I. P. Elliott, S. B. Duckett, G. G. R. Green, I. G. Khazal, J. López-Serrano, D. C. Williamson, Reversible interactions with para-hydrogen enhance NMR sensitivity by polarization transfer. Science 323, 1708–1711 (2009). 19325111 10.1126/science.1168877

[19] M. Suefke, S. Lehmkuhl, A. Liebisch, B. Blumich, S. Appelt, Para-hydrogen raser delivers sub-millihertz resolution in nuclear magnetic resonance. Nat. Phys. 13, 568–572 (2017).

[20] A. N. Pravdivtsev, F. D. Sönnichsen, J. B. Hövener, Continuous radio amplification by stimulated emission of radiation using parahydrogen induced polarization (PHIP-RASER) at 14 Tesla. Chem. Phys. Chem. 21, 667–672 (2020). 31898393 10.1002/cphc.201901056 PMC7187451

[21] B. Joalland, N. M. Ariyasingha, S. Lehmkuhl, T. Theis, S. Appelt, E. Y. Chekmenev, Parahydrogen-induced radio amplification by stimulated emission of radiation. Angew. Chem. Int. Ed. 59, 8654–8660 (2020). 10.1002/anie.201916597 PMC7437572 32207871

[22] P. T. Callaghan, *Principles of Nuclear Magnetic Resonance Microscopy* (Clarendon Press, 1993).

[23] P. C. Lauterbur, P. Mansfield, *The Nobel Prize in Physiology or Medicine* (2003); www.nobelprize.org/prizes/medicine/2003/summary/.

[24] A. Vlassenbroek, J. Jeener, P. Broekaert, Radiation damping in high resolution liquid NMR: A simulation study. J. Chem. Phys. 103, 5886–5897 (1995).

[25] Y. Y. Lin, N. Lisitza, S. Ahn, W. S. Warren, Resurrection of crushed magnetization and chaotic dynamics in solution NMR spectroscopy. Science 290, 118–121 (2000). 11021793 10.1126/science.290.5489.118

[26] M. T. Pöschko, V. V. Rodin, J. Schlagnitweit, N. Müller, H. Desvaux, Nonlinear detection of secondary isotopic chemical shifts in NMR through spin noise. Nat. Commun. 8, 13914 (2017). 28067218 10.1038/ncomms13914 PMC5227550

[27] N. Müller, A. Jerschow, Nuclear spin noise imaging. Proc. Natl. Acad. Sci. U.S.A. 103, 6790–6792 (2006). 16636281 10.1073/pnas.0601743103 PMC1458973

[28] M. Suefke, A. Liebisch, B. Blümich, S. Appelt, External high-quality-factor resonator tunes up nuclear magnetic resonance. Nat. Phys. 11, 767–771 (2015).

[29] M. P. Augustine, S. D. Bush, E. L. Hahn, Noise triggering of radiation damping from the inverted state. Chem. Phys. Lett. 322, 111–118 (2000).

[30] A. Jurkiewicz, Properties and edition of NMR spontaneous maser emission spectra. Appl. Magn. Reson. 50, 709–724 (2019).

[31] X. A. Mao, C. H. Ye, Understanding radiation damping in a simple way. Concept. Magn. Reson. 9, 173–187 (1997).

[32] M. P. Augustine, Transient properties of radiation damping. Prog. Nucl. Magn. Reson. Spectrosc. 40, 111–150 (2002).

[33] V. V. Krishnan, N. Murali, Radiation damping in modern NMR experiments: Progress and challenges. Prog. Nucl. Magn. Reson. Spectrosc. 68, 41–57 (2013). 23398972 10.1016/j.pnmrs.2012.06.001 PMC3644564

[34] S. H. Strogatz, From Kuramoto to Crawford: Exploring the onset of synchronization in populations of coupled oscillators. Physica D 143, 1–20 (2000).

[35] Y. Kuramoto, D. Battogtokh, Coexistence of coherence and incoherence in nonlocally coupled phase oscillators. Nonlinear Phenom. Complex Syst. 5, 380 (2002).

[36] Y. Kuramoto, H. Nakao, On the concept of dynamical reduction: The case of coupled oscillators. Philos. Trans. Royal Soc. A 377, 20190041 (2019). 10.1098/rsta.2019.0041 PMC6834004 31656146

[37] X. A. Mao, C. H. Ye, Line shapes of strongly radiation-damped nuclear magnetic resonance signals. J. Chem. Phys. 99, 7455–7462 (1993).

[38] X. A. Mao, J. X. Guo, C. H. Ye, Nuclear-magnetic-resonance line-shape theory in the presence of radiation damping. Phys. Rev. B 49, 15702–15711 (1994). 10.1103/physrevb.49.15702 10010702

[39] D. J. Y. Marion, P. Berthault, H. Desvaux, Spectral and temporal features of multiple spontaneous NMR-maser emissions. Eur. Phys. J. D 51, 357–367 (2009).

[40] V. Henner, H. Desvaux, T. Belozerova, D. J. Y. Marion, P. Kharebov, A. Klots, Collective effects due to dipolar fields as the origin of the extremely random behavior in hyperpolarized NMR maser: A theoretical and numerical study. J. Chem. Phys. 139, 144111 (2013). 24116607 10.1063/1.4823823

[41] M. J. Cowley, R. W. Adams, K. D. Atkinson, M. C. R. Cockett, S. B. Duckett, G. G. R. Green, J. A. B. Lohman, R. Kerssebaum, D. Kilgour, R. E. Mewis, Iridium N-heterocyclic carbene complexes as efficient catalysts for magnetization transfer from para-hydrogen. J. Am. Chem. Soc. 133, 6134–6137 (2011). 21469642 10.1021/ja200299u PMC3080106

[42] S.-C. Lee, K. Kim, J. Kim, S. Lee, J. H. Yi, S. W. Kim, K.-S. Ha, C. Cheong, One micrometer resolution NMR microscopy. J. Magn. Reson. 150, 207–213 (2001). 11384182 10.1006/jmre.2001.2319

[43] L. Ciobanu, D. A. Seeber, C. H. Pennington, 3D MR microscopy with resolution 3.7 microm by 3.3 microm by 3.3 microm. J. Magn. Reson. 158, 178–182 (2002). 12419685 10.1016/s1090-7807(02)00071-x

[44] A. J. Ilott, A. Jerschow, Super-resolution surface microscopy of conductors using magnetic resonance. Sci. Rep. 7, 5425 (2017). 28710421 10.1038/s41598-017-05429-3 PMC5511221

[45] J. Schlagnitweit, S. W. Morgan, M. Nausner, N. Müller, H. Desvaux, Non-linear signal detection improvement by radiation damping in single-pulse NMR spectra. Chem. Phys. Chem. 13, 482–487 (2012). 22266720 10.1002/cphc.201100724 PMC3307601

[46] B. Joalland, T. Theis, S. Appelt, E. Y. Chekmenev, Background-free proton NMR spectroscopy with radiofrequency amplification by stimulated emission radiation. Angew. Chem. Int. Ed. 60, 26298–26302 (2021). 10.1002/anie.202108939 PMC8629966 34459080

[47] J. De Wilde, D. Grainger, D. Price, C. Renaud, Magnetic resonance imaging safety issues including an analysis of recorded incidents within the UK. Prog. Nucl. Magn. Reson. Spectrosc. 51, 37–48 (2007).

[48] S. Kaka, M. R. Pufall, W. H. Rippard, T. J. Silva, S. E. Russek, J. A. Katine, Mutual phase-locking of microwave spin torque nano-oscillators. Nature 437, 389–392 (2005). 16163351 10.1038/nature04035

[49] C. Bick, M. Goodfellow, C. R. Laing, E. A. Martens, Understanding the dynamics of biological and neural oscillator networks through exact mean-field reductions: A review. J. Math. Neurosci. 10, 9 (2020). 32462281 10.1186/s13408-020-00086-9 PMC7253574

[50] J. T. Vaughan, M. Garwood, C. M. Collins, W. Liu, L. D. Barre, G. Adriany, P. Andersen, H. Merkle, R. Goebel, M. B. Smith, K. Ugurbil, 7T vs. 4T: RF power, homogeneity, and signal-to-noise comparison in head images. Magn. Reson. Med. 46, 24–30 (2001). 11443707 10.1002/mrm.1156

[51] F. Engelke, Virtual photons in magnetic resonance. Concepts Magn. Reson. Part A 36A, 266–339 (2010).

[52] X. A. Mao, Calculation of the energy transferred by radiation damping from nuclear spin system to receiver coil in NMR. Chem. Phys. Lett. 756, 137853 (2020).

[53] S. Nadis, All together now. Nature 421, 780–782 (2003). 12594475 10.1038/421780a

[54] P. Ebrahimzadeh, M. Schiek, P. Jaros, T. Kapitaniak, S. van Waasen, Y. Maistrenko, Minimal chimera states in phase-lag coupled mechanical oscillators. Eur. Phys. J. Spec. Top. 229, 2205–2214 (2020).

[55] A. Ruotolo, V. Cros, B. Georges, A. Dussaux, J. Grollier, C. Deranlot, R. Guillemet, K. Bouzehouane, S. Fusil, A. Fert, Phase-locking of magnetic vortices mediated by antivortices. Nat. Nanotechnol. 4, 528–532 (2009). 19662017 10.1038/nnano.2009.143

[56] E. N. Lorenz, Deterministic nonperiodic flow. J. Atmos. Sci. 20, 130–141 (1963).

