# Supplementary material for: RASER MRI: Magnetic resonance images formed spontaneously exploiting cooperative nonlinear interaction
Source: Sci Adv. 2022 Jul 13;8(28):eabp8483. doi: 10.1126/sciadv.abp8483 (PMC9278855; doi:10.1126/sciadv.abp8483)
Supplement: Supplementary file 1 — Supplementary Text Figs. S1 to S12 References [file sciadv.abp8483_sm.pdf]

Science Advances
AAAS

Supplementary Materials for

RASER MRI: Magnetic resonance images formed spontaneously exploiting cooperative nonlinear interaction

Sören Lehmkuhl *et al.*

Corresponding author: Sören Lehmkuhl, lehmkuhl@kit.edu; Thomas Theis, ttheis@ncsu.edu;
Stephan Appelt, st.appelt@fz-juelich.de

Sci. Adv. **8**, eabp8483 (2022)
DOI: 10.1126/sciadv.abp8483

This PDF file includes:

Supplementary Text
Figs. S1 to S12
References

Supplementary Text

This supplement discusses the theory and the physics of RASER modes in the presence of a magnetic field gradient. It aims to explain many phenomena for RASER MRI in one dimension. For this purpose the supplement is subdivided into six subsections. We first introduce a model to extend the nonlinear multi-mode RASER theory (*12*, *16*), which describes *N* nonlinear interacting RASER modes (*9*, *15*, *20*). Section 1 describes the derivation of the equations of motion governing RASER MRI in one dimension. A spatial encoding procedure is added, which divides the imaging domain $\Delta = \gamma_H G_z$ L in *N* nonlinear interacting slices, where γ_H, G_z and L are the ^{1}H gyromagnetic ratio, the magnetic field gradient dB_0/dz, and the sample extension, respectively. In section 2, we derive the point spread function (PSF) for one single RASER mode or slice ($N = 1$) excluding T_1 relaxation: The hyperbolic secant. In section 3, T_1 relaxation is included, and it is shown that the corresponding PSF as a function of time is an asymmetric, distorted hyperbolic secant. In section 4, RASER MRI simulations for $N > 20$ interacting slices are discussed, especially for a rectangular profile (4a), a rectangular profile superimposed by a sinusoidal modulation (4b) and for a profile close to our experimental two-chamber setup (4c). Section 5 analyzes the artifacts shown in the RASER images of the main text. In section 6, three invariance principles for RASER MRI are introduced together with a discussion of their consequences. Finally, section 7 concludes with a discussion of the relation between RASER MRI and other research fields.

1. Theory of one-dimensional RASER imaging with *N* interacting modes

As a starting point, one dimensional imaging is discussed. In this case, only one magnetic field gradient G_z = dB_0/dz is applied and the resulting images are one-dimensional projections. We assume that the sample has been polarized into a state of negative spin polarization. In this manuscript, the sample is pumped by SABRE, but nothing precludes the use of other hyperpolarization techniques. We additionally assume that there is no additional pumping (the pumping period is over) and there was a small but sufficient waiting time to assure that there is no more movement in the sample (see Fig. 2, main text). This will be the initial time at $t = 0$ which is characterized by a total population inversion d_0. In a one-dimensional model, the sample is characterized by its extension *L* along the z-direction, a center z_0 and two boundaries at positions z_0 + L/2 and z_0 – L/2, as shown in Fig. S1(A).

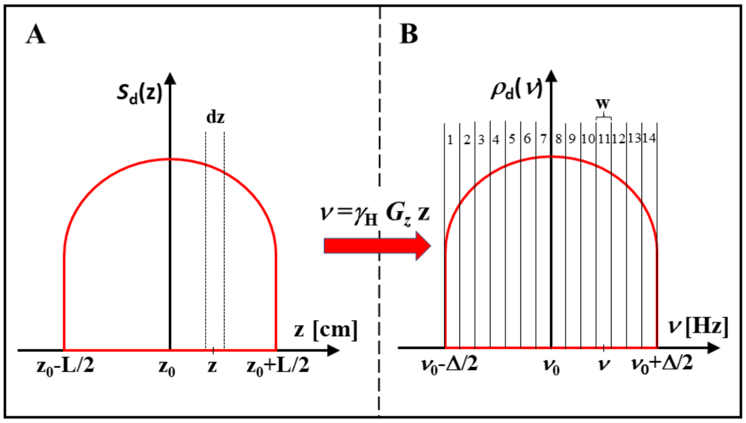

Fig. S1. Transformation of the imaging profile from space into the frequency domain. (**A**) Example of a one-dimensional image with a hemicircular shaped top, where S_d(z)dz, the number of inverted spins per slice thickness dz at position z, is plotted against the spatial z-coordinate. The total extension of the sample is L, z_0 denotes the center, the left and right boundaries are located at z_0 – L/2 and z_0 + L/2. (**B**) Given a gradient G_z the density S_d(z) transforms into the density $\rho_d(\nu)$ after a linear transformation $\nu = \gamma_H G_z$ z is performed on the z-axis. $\rho_d(\nu)$dν is defined as the number of inverted spins at frequency ν in the interval dν. The center frequency of the sample is $\nu_0 = \gamma_H B_0/2\pi$, the left and right boundaries are located at ν_0–Δ/2 and ν_0+Δ/2, where the image domain is defined as $\Delta = \gamma_H G_z$ L. Here the image is divided into N = 14 slices, where the width of each slice is given by the line-width w = 1/(πT_2^*).

If the distribution of the population inversion at position z is the one-dimensional density function $S_d(z)$, then $S_d(z)dz$ is the number of negatively polarized spins in the spatial interval dz at the location z. In the presence of the gradient G_z the z-dimension is transformed into the frequency space according to a linear transformation $\nu = \gamma_H G_z z$. After this linear transformation, the density $S_d(z)$ transforms into the one-dimensional density $\rho_d(\nu)$, where $\rho_d(\nu)d\nu$ is the number of negatively polarized spins at the frequency interval $[\nu,\nu+d\nu]$. The function $\rho_d(\nu)$ is shown on the right side of Fig. S1 with a center frequency ν_0 and the two boundaries at $\nu_0+\Delta/2$ and $\nu_0-\Delta/2$. The image domain in frequency space is given by $\Delta = \gamma_H G_z L$. Given the two density functions $S_d(z)$ and $\rho_d(\nu)$ in the spatial and frequency domain, the total initial population inversion d_0 of the sample is given by the integral

$$d_0 = \int_{z_0-L/2}^{z_0+L/2} S_\mathrm{d}(z)\,\mathrm{d}z \ = \int_{\nu_0-\Delta/2}^{\nu_0+\Delta/2} \rho_\mathrm{d}(\nu)\,\mathrm{d}\nu \qquad \text{(S1).}$$

An assumption made for the simulations of RASER MRI is that RASER activity in the presence of G_z is self-organized. Thus, $N >>1$ modes or slices are introduced to enable numerical simulations of the theory. A good estimate for the number of slices is given by $N = \Delta/\delta\nu$, where the slice separation is chosen $\delta\nu < w$. We found by numerical evaluation that if $\delta\nu < w$, the properties and the shape of the resulting RASER images do not change (invariance principle I). For a simulation of a RASER image without numerical artifacts in the image domain $\Delta = \gamma_H G_z L$, a division into $N = \Delta/\delta\nu \sim\Delta/w_{as}$ slices is sufficient, where $w_{as} \sim 1/T_1$ is the width of the asymmetric point spread function of a single RASER mode (see section 3). Each RASER-active slice with label $\mu = 1..N$ can be attributed to a center frequency ν_μ given by

$$\nu_\mu = \nu_0 - \frac{1}{2}[\Delta - (2\mu - 1)\delta\nu], \mu = 1..N \qquad \text{(S2).}$$

The ensemble RASER image can be described by a superposition of $N = \Delta/\delta\nu$ slices. In contrast to regular MRI with its Lorentzian shaped PSFs, the spectrum associated to each slice is very complex, because all slices are all coupled between each other. This is shown in section 4(a).

In the following we demonstrate the essential steps leading to the RASER MRI equations, which describe the dynamics of all $\mu = 1..N$ RASER slices. We start with the multi-mode RASER theory as described previously (*12,16*). These are given by a set of $2N$ non-linear coupled differential equations for N modes. The dynamic of each mode or of the spin species with angular frequency ω_μ is described by the population inversion d_μ and the complex-valued transverse spin component $\alpha_\mu = A_\mu \exp(i\phi_\mu)$, where A_μ and ϕ_μ are the amplitude and phase of each mode, respectively (*12*):

$$\dot{d}_\mu = \Gamma_\mu(d_{\mu,0} - d_\mu) - \frac{d_\mu}{T_1} - 2\beta \sum_{\nu,\sigma=1}^{N} \{\alpha_\nu\alpha_\sigma^* + \alpha_\nu^*\alpha_\sigma\} \qquad \text{(S3),}$$

$$\dot{\alpha}_\mu = \left\{-\frac{1}{T_2^*} + i\omega_\mu\right\}\alpha_\mu + \beta d_\mu \sum_{\tau=1}^{N} \alpha_\tau \qquad \text{(S4).}$$

The form of these equations, which is formulated in the rotating frame, are based on the multi-mode LASER equations, as published by H. Haken (*15*), and by applying the adiabatic elimination of fast variables (the slavery principle) to the fast-decaying electromagnetic field modes of the LC resonator (*12*). For one single mode ($\mu = 1$) the form of Eqs.(S3,S4) is identical to the extended Bloch equations for the longitudinal and transverse magnetization M_z and M_T, respectively, used in many radiation damping studies (*4, 24, 31, 32, 38, 39, 52*). The magnetizations for one mode are related to the variables d_1 and α_1 through $M_z = -(\hbar\gamma_H/2V_S)d_1$ and $M_T = -(\hbar\gamma_H/V_S)\alpha_1$, respectively.

The coupling parameter in Eqs.(S3,S4) is given by $\beta = \mu_0\, \hbar\, \gamma_H^2\, Q/(4V_s)$, where μ_0, $\hbar$ and γ_H denote the vacuum permeability, Planck's reduced constant and the ^1H gyromagnetic ratio. V_s is the sample volume and Q the quality factor of the resonator, respectively. The coupling constant β is related to the resonator damping rate $\kappa_m = \omega_0/Q$ and to the magnetic coupling constant $|g_m|^2 = \gamma_H^2\, \mu_0\, \hbar\, \omega_0/(4V_s)$ by $\beta = |g_m|^2/\kappa_m$. The factors $1/T_1$ and $1/T_2^*$ represent the longitudinal and effective transverse relaxations rates. Each population inversion d_μ of mode μ in Eq.(3) is pumped with the rate Γ_μ towards the equilibrium population inversion $d_{\mu,0}$, decays with the rate $1/T_1$ and is diminished by the last term on the right side of Eq.(S3), which depends on the sum over all quadratic terms $\alpha_\nu\, \alpha_\sigma^* + \alpha_\nu^*\alpha_\sigma$. The transverse modes α_μ in Eq.(S4) decay with the rate $1/T_2^*$ and oscillate with the off-resonance angular frequency ω_μ. For NMR spectroscopy the origin of these frequencies could be different chemical shifts or splittings due to *J*-coupling. The last term in Eq.(S4), proportional to the term $\beta\, d_\mu\, \Sigma\alpha_\tau$, is the source for the RASER emission of mode μ and is responsible for collective phenomena. The term $\beta\, d_\mu\, \alpha_\mu$ for $\tau = \mu$ is responsible for RASER emission. The product $\beta\, d_\mu = \mu_0\, \hbar\, \gamma_H^2\, Q\, d_\mu/(4V_s)$ can be described as radiation de-damping or damping terms of mode μ, depending on whether the sign of d_μ is positive or negative, respectively. This is analogous to radiation damping described in the modified Bloch equations (*4, 24, 31, 32, 38, 39, 52*). The cross terms, $\beta\, d_\mu\, \alpha_\tau$ for $\tau \neq \mu$, are not included in the extended Bloch equations, but are essential to describe the dynamics of RASER MRI. Their sum determines the time evolution of each of the amplitudes A_μ and phases ϕ_μ. This has various consequences for RASER images, such as edge artefacts, and non-linear amplitude deformations, as will be shown later.

The RASER MRI equations for one ^1H spin species can be derived based on Eqs.(S3,S4): First, the spin system is pumped into a state with highly negative ^1H polarization P_H, i.e. into a state with a large total population inversion d_0 (see Eq.(S1)). For ^1H SABRE pumped organic molecules, such as pyrazine or pyridine, the proton polarization is typically in the range from $P_H \sim -10^{-3}$ up to -10^{-1}, which for our experimental conditions corresponds to values $d_0 \sim 10^{16}$-10^{18} (for calculation see Materials and Methods section). To prevent any RASER emission, a strong crusher gradient or a detuned resonance LC circuit ensure that $\alpha_\mu = 0$ during the pumping. Thus, only the pumping and T_1 relaxation terms on the right side of Eq.(S3) are relevant during the pumping period. Second, after the pumping and the strong crusher gradient is switched off (all $\Gamma_\mu = 0$), and provided the weak gradient G_z for imaging is not too strong, some slices start to be RASER active and oscillate in the presence of G_z. The angular frequency $\omega_\mu = 2\pi\nu_\mu$ of each RASER active slice is given by Eq.(S2). After splitting Eq.(S4) into a real part describing the amplitudes A_μ and an imaginary part, which describes the phases ϕ_μ, a set of $3N$ nonlinear coupled differential equations for the variables d_μ, A_μ and ϕ_μ is obtained. The corresponding model for RASER MRI is given by Eqs.(S5-S8).

Eq.(S8) is a boundary condition which defines the initial population inversion $d_\mu(0)$. The presence of the weak gradient G_z separates the total population inversion d_0 into $N = \Delta/\delta\nu$ slices, where the initial value of $d_\mu(0)$ for each slice is given by Eq.(S8), namely the integral of $\rho_d(\nu)$ over the frequency range of slice μ with boundaries $[\nu_0 - \Delta/2 + (\mu + 1)\delta\nu\, ,\ \nu_0 - \Delta/2 + \mu\, \delta\nu]$. The density $\rho_d(\nu)$ can be seen as a given input profile after pumping of the sample, which depends on the shape of the imaged object.

$$\dot{d}_{\mu} = -\frac{d_{\mu}}{T_1} - 4\beta \sum_{\sigma,\tau=1}^{N} A_{\sigma} A_{\tau} \cos(\phi_{\sigma} - \phi_{\tau}) \quad \text{(S5)},$$

$$\dot{A}_{\mu} = -\frac{A_{\mu}}{T_2^*} + \beta d_{\mu} \sum_{\tau=1}^{N} A_{\tau} \cos(\phi_{\tau} - \phi_{\mu}) \quad \text{(S6)},$$

$$\dot{\phi}_{\mu} = 2\pi\{\nu_0 - 0.5[\Delta - \delta\nu(2\mu - 1)]\} + \beta \frac{d_{\mu}}{A_{\mu}} \sum_{\tau=1}^{N} A_{\tau} \sin(\phi_{\tau} - \phi_{\mu}) \quad \text{(S7)},$$

$$d_{\mu}(0) = \int_{\nu_0 - \Delta/2 + (\mu-1)\delta\nu}^{\nu_0 - \Delta/2 + \mu\delta\nu} \rho_{\mathrm{d}}(\nu)\, d\nu \quad \text{(S8)}.$$

Given $\rho_{\mathrm{d}}(\nu)$, the quantities d_{μ}, A_{μ} and ϕ_{μ} can be evaluated by numerical evaluation of Eqs.(S5-S7) with the given boundary condition Eq.(S8), and the measurable total transverse RASER signal results as a superposition of all functions $\alpha_{\mu} = A_{\mu} \exp(\mathrm{i}\phi_{\mu})$.

$$Sig(t) = \frac{1}{\sqrt{N}} \sum_{\mu=1}^{N} A_{\mu}(t)\, \mathrm{Re}\{\exp[\,i\phi_{\mu}(t)]\} \quad \text{(S9)}.$$

The Fourier transformation of the total signal *Sig*(t) Eq.(S9) results in the RASER image. Note that no boundary conditions for the initial amplitudes $A_{\mu}(0)$ and phases $\phi_{\mu}(0)$ are given in Eq.(S8). The reason is that the resulting RASER images are practically independent from the initial values of $A_{\mu}(0)$ and $\phi_{\mu}(0)$, no matter whether $A_{\mu}(0)$ and $\phi_{\mu}(0)$ are random or defined values. Provided the condition $T_1 >> T_2^*$ holds (which is mostly the case), extensive numerical simulations reveal three invariance principles with respect to the Fourier transformed RASER images in the absolute mode:

I. The RASER image does not depend on to the slicing $\delta\nu$ as long as $\delta\nu$ < w. II. The RASER image contrast and resolution is independent of T_1, and III. The RASER image is invariant with respect to the initial conditions $A_{\mu}(0)$ and $\phi_{\mu}(0)$. These three invariance principles have significant consequences for RASER MRI.

The invariance from the choice of the slicing distance $\delta\nu$ (principle I) means that a continuous limit $N \to \infty$ exists, where the discrete variables $d_{\mu}(t)$, $A_{\mu}(t)$ and $\phi_{\mu}(t)$ become continuous variables. In this limit all sums in Eqs.(S5-S8) become integrals. Another consequence is that numerical simulations produce reliable results without changing the involved physics as long as $\delta\nu$ < w. We found that ripple-like artifacts arise in the image if $\delta\nu$ ~ w, but the envelope of the image is the same as $\delta\nu$ << w. However, if $\delta\nu$ << w, the numerical simulations can become very time-consuming. A good compromise is $\delta\nu \approx 1/T_1 \approx \mathrm{w}_{\mathrm{as}}$, where no ripples are visible.

The invariance of the RASER image shape and contrast on the value of T_1 (principle II) means that the contrast of RASER MRI cannot be associated to the width of single PSFs. This includes the width of the asymmetric PSF $\mathrm{w}_{\mathrm{as}} \approx 1/T_1$ of a single RASER mode, introduced in section 3. The contrast mechanism is based on collective interactions, as will be shown in chapter 4(a).

The invariance of the RASER image on the initial conditions $A_{\mu}(0)$ and $\phi_{\mu}(0)$ (principle III) allows for reproducible results irrespective of the noise excitation. The amplitude and shape of the RASER image do

not change if the RASER burst is initiated either by spin noise or a defined excitation sequence with a weak RF or DC pulse. We found that for very different initial conditions $A_\mu(0)$, $\phi_\mu(0)$ (more than one order of magnitude), there is a small shift of the entire RASER burst signal. This leaves the absolute Fourier transformed spectrum invariant but the phased spectrum is shifted by a global phase. This small shift can be avoided by applying a weak DC or RF pulse to initiate the reproducible RASER bursts, beneficial for averaging and 2D RASER MRI.

A detailed mathematical analysis of the dynamics of the image formation for arbitrary input profiles $\rho_d(\nu)$ and how to explain the three invariance principles is quite elaborate and not the main focus of this contribution. Therefore, we focus on numerical simulations to investigate image artifacts and the more sensitive contrast mechanism. In section 4(a) we use the simple example of a rectangular profile. Due to the invariance principle III, we chose the simplest case, $\phi_\mu(0) = 0$ and a constant small value $A_\mu(0) \sim 10^9$–10^{10}. The value for $A_\mu(0)$ is in the order of the spin noise amplitude $N_s^{1/2}$ (*27*), where $N_s \sim 10^{19}$-10^{20} is the total number of spins in the sample.

Finally, we analyze whether a local threshold condition exists for RASER MRI. Without gradient**,** there is only one RASER mode (N = 1). The threshold condition for RASER action is $d_0 \geq d_{\mathrm{th}} = 4V_s/(\mu_0 \hbar \gamma_{\mathrm{H}}^2\, T_2^* Q)$,(*12*) where d_{th} is the threshold population inversion. An equivalent expression for this threshold condition is $\varepsilon = d_0/d_{\mathrm{th}} = T_2^*/\tau_{\mathrm{rd}} \geq 1$, where ε is a dimensionless quantity characterizing the threshold. However, if a gradient is applied, the sample divides into $N = \Delta/\delta\nu >> 1$ slices and the threshold condition $\varepsilon = d_0/d_{\mathrm{th}} > 1$ cannot be applied anymore as a proper criterion for RASER activity.

When a gradient is applied, the population inversion d_0 is distributed over the image domain Δ according to the population inversion density $\rho_d(\nu)$. Assuming no interaction between the slices $\delta\nu$, a threshold condition can be formulated based on the population inversion within a frequency interval $\mathrm{w} = 1/(\pi T_2^*)$. Within this frequency interval the damping with rate $1/T_2^*$ is compared to the RASER dedamping process. A region $r_\mu = [\nu_\mu - \mathrm{w}/2,\ \nu_\mu + \mathrm{w}/2]$ at position ν_μ and width $\mathrm{w} = 1/(\pi T_2^*)$ is RASER active if $\int_{\nu_\mu - \mathrm{w}/2}^{\nu_\mu + \mathrm{w}/2} \rho_{\mathrm{d}}(\nu)\, d\nu \geq d_{\mathrm{th}}$. One simple example is the case of a rectangular profile, where $\rho_d(\nu) = \rho_d^{\mathrm{rect}} = d_0/\Delta$. Now, the integral becomes $\int_{\nu_\mu - \mathrm{w}/2}^{\nu_\mu + \mathrm{w}/2} \rho_{\mathrm{d}}(\nu)\, d\nu = \rho_{\mathrm{d}}^{\mathrm{rect}} \cdot \mathrm{w} = d_0 \mathrm{w}/\Delta$, and the above threshold condition reduces to $d_0 \mathrm{w}/\Delta \geq d_{\mathrm{th}}$. This expression can be written as $\rho_d^{\mathrm{rect}} \geq \rho_d^{\mathrm{th}}$, where $\rho_d^{\mathrm{th}} = d_{\mathrm{th}}/\mathrm{w}$ is the threshold population inversion density. Consequently, if $\rho_d^{\mathrm{rect}} < \rho_d^{\mathrm{th}}$, no RASER action should be possible for any region r_μ.

Unfortunately, this condition is not sufficient as a strict threshold condition in the presence of nonlinear coupling. Numerical simulations show that if ρ_d^{rect} is slightly below ρ_d^{th} a region r_μ in the center of Δ can be RASER active while the regions at the boundaries are not. Close to the center the slices cooperate with all their neighbors in a constructive way, while the slices close to the two boundaries cooperate destructively with their neighbors. Because of the cooperative action between slice μ with all other slices, a local threshold condition does not exist and has to be replaced by a non-local threshold condition. Numerical simulations show that RASER action for a region r_μ in the domain Δ depends on d_0, on the width of the image domain Δ and on the detailed shape of $\rho_d(\nu)$. A detailed mathematical evaluation of this statement is quite elaborate, but a numerical evaluation of the case using a rectangular profile is shown in section 4(a). In nearly all simulations shown in the following the threshold population inversion density $\rho_d^{\mathrm{th}} = 4\pi V_{\mathrm{s}}/\left(\mu_0 \hbar \gamma_{\mathrm{H}}^2 Q\right)$ is used as a reference for RASER activity (even if not strictly valid) and indicated by a dashed green line.

A maximum gradient $G_{\max}$ for RASER activity can be deduced from the threshold condition $\rho_{\mathrm{d}}^{\mathrm{rect}} = d_0/\Delta = \rho_{\mathrm{d}}^{\mathrm{th}}$ and in the absence of nonlinear coupling. From $\Delta = \gamma_{\mathrm{H}}\, G_{\max}\, \mathrm{L}$ and $\rho_d^{\mathrm{th}} = 4\pi V_{\mathrm{s}}/\left(\mu_0 \hbar \gamma_{\mathrm{H}}^2 Q\right)$ we obtain the maximum gradient $G_{\max} = \mu_0 \hbar \gamma_{\mathrm{H}} d_0 Q/(4\pi V_s L)$. This means that $G_{\max}$ is large for high Q resonators, large values of d_0 and if the sample is small.

2. RASER with N = 1, Point Spread Function neglecting T_1 relaxation

We will proceed with the simplest possible case, and derive the exact solution for one RASER mode ($N = 1$) if T_1 relaxation can be completely neglected ($T_1 = \infty$). In this case an exact solution in the form of tanh- and sech-functions exists. The relevance for the line shape in NMR spectroscopy due to strong radiation damping effects has been demonstrated (*4, 24, 31, 32, 37-39, 52*). Here, we repeat the derivation of the basic results described there for two reasons. The first reason is to be compatible with the nomenclature used here. Secondly, Mao *et al.* (*31, 37, 38*) studied amongst others the radiation damped signal burst after a radio frequency pulse excitation angle close to π, which is in close correspondence to one self-induced RASER burst in Eqs.(S5-S8). For $N = 1$ and neglecting T_1 relaxation Eqs.(S5-S8) are reduced to their most basic form,

$$\dot{d} = -4\beta A^2 \qquad \text{(S10)},$$

$$\dot{A} = \left(\beta d - \frac{1}{T_2^*}\right) A \qquad \text{(S11)},$$

$$\dot{\phi} = \omega_1 \qquad \text{(S12)}.$$

We set the boundary conditions for Eqs.(S10-S12) as $\phi(0) = 0, A(0) = A_0 = 10^{12} << d_0, d(0) = d_0$. The two Eqs.(S11,S12) are equivalent to one single differential equation for the complex-valued transverse spin component $\alpha(t) = A(t)\exp(i\omega_1 t)$, given by $\dot{\alpha} = (\beta d - 1/T_2^* + i\omega_1)\alpha$. The derivation of the exact solution for $d(t)$ and $A(t)$ follows from differentiation of Eq.(S10), which results in $\partial^2 d/\partial t^2 = -8\beta A \cdot \partial A/\partial t$. After substituting the right side of Eq.(S11) for $\partial A/\partial t$ in the preceding expression we obtain

$$\frac{\partial^2 d}{\partial t^2} = -8\beta A \cdot \left[\beta A \cdot d - \frac{A}{T_2^*}\right] \underbrace{=}_{\text{Eq.(S10)}} \left(2\beta d - \frac{2}{T_2^*}\right) \cdot \frac{\partial d}{\partial t} \qquad \text{(S13)}.$$

Setting $U = \partial d/\partial t$, Eq.(S13) turns into $\partial U/\partial t = (2\beta d - 2/T_2^*)\,\partial d/\partial t$, which is equivalent to $\partial U/\partial t = 2\beta d - 2/T_2^*$. The solution is given by integration, which results in $U = \partial d/\partial t = \beta d^2 - (2/T_2^*)d + C$. The integration constant C is fixed by the initial conditions $A(0) \approx 0 << d_0$, $d(0) = d_0$, so $dd/dt = -4\beta A^2(0) \approx 0$, giving $C = d_0(2/T_2^* - \beta d_0)$. After introducing the parameter $q = (1 + \varepsilon^2 - 2\varepsilon)^{1/2}$, where the dimensionless variable is $\varepsilon = T_2^*/\tau_{rd} = T_2^* \beta d_0$, the previous equation for U can be written as

$$U = \frac{\partial d}{\partial t} = \beta \left[-\left(\frac{q}{\beta T_2^*}\right)^2 + \left(d - \frac{1}{\beta T_2^*}\right)^2\right] \qquad \text{(S14)}.$$

Introducing the parameters $a = 1/(T_2^*\beta)$ and $b = q/(T_2^*\beta)$, Eq.(S14) simplifies to $\partial d/\partial t = \beta\,[-b^2 + (d - a)^2]$, which is equivalent to

$$\frac{1}{\beta}\int\frac{\partial d}{(d-\mathrm{a})^2-\mathrm{b}^2}=\int\partial t=(t-t_0) \qquad \text{(S15).}$$

This integral is known as $\int dx/[(\mathrm{a}-x)^2-\mathrm{b}^2]=\mathrm{b}^{-1}\,\mathrm{arctanh}[(\mathrm{a}-x)/\mathrm{b}]$, so Eq.(S15) can be written as $\mathrm{arctanh}[(1-\beta T_2^* d)/q]=(t-t_0)q/T_2^*$. After isolating *d* on the left side we get

$$d=\frac{1}{\beta T_2^*}\left\{1-q\,\tanh\left[\frac{q}{T_2^*}(t-t_0)\right]\right\} \qquad \text{(S16).}$$

The exact form for the transverse spin component $A(t)$ can be derived by differentiation of Eq.(S16). Using the relation $\partial\tanh(x)/\partial x=1\text{-}\tanh^2(x)=\mathrm{sech}^2(x)$ this leads to

$$\frac{\partial d}{\partial t}=\frac{-1}{\beta T_2^*}\frac{q}{T_2^*}q\left\{1-\tanh^2\left[\frac{q}{T_2^*}(t-t_0)\right]\right\} \qquad \text{(S17).}$$

According to Eq.(S10) $\partial d/\partial t=\text{-}\,4\beta A^2$ so Eq.(S17) can also be written as $A^2=\beta^{-2}(q/2T_2^*)^2\,\mathrm{sech}^2[q(t-t_0)/T_2^*]$. Applying the square root on both sides of the preceding equation, *A* is finally

$$A=\frac{q}{2\beta T_2^*}\mathrm{sech}\left[\frac{q}{T_2^*}(t-t_0)\right] \qquad \text{(S18).}$$

According to Eq.(S18), *A* is a hyperbolic secant function (soliton solution in the time domain) and is symmetric with respect to t_0, which is the time of maximum amplitude. An interesting feature of Eq.(S18) is that the envelope of the Fourier transformed spectrum in the frequency domain ω is once again a sech-function. According to Mao *et al*. (*37, 38*) this envelope is modulated by a phase factor $\cos(\omega t_0)$, i.e.

$$S(\omega)=\frac{\pi}{2\beta}\frac{q}{T_2^*}\mathrm{sech}\left[\frac{\pi T_2^*\omega}{2q}\right]\cos[\omega t_0] \qquad \text{(S19).}$$

The Fourier transformed spectrum of the transverse spin component $\alpha = A\exp(\mathrm{i}\omega_1 t)$ is obtained from Eq.(S19) simply by replacing the angular frequency ω in the argument of the sech function and in the cos term by ω - ω_1 (Fourier shift theorem). By inspection of Mao *et al*. (*37, 38*) it can be shown that for the case of a self-induced RASER burst, the time t_0 is given by

$$t_0=-\frac{T_2^*}{q}\mathrm{arctanh}\left[\frac{1+\varepsilon\cos\theta_0}{q}\right] \qquad \text{(S20).}$$

The exact expression for the factor is $q = \sqrt{1 + \varepsilon^2 + 2\varepsilon \cos\theta_0}$, $\varepsilon = T_2^*/\tau_{rd} = T_2^*\beta d_0$ and the initial flip angle is given by $\theta_0 = \pi - 2\arcsin(A(0)\beta T_2^*/\varepsilon)$. The initial flip angle θ_0 after applying RF-pulses close to 180°, as discussed by Mao *et al*., is replaced for the RASER by a small initial fluctuation of the transverse spin component $A(0) = A_0$ at time $t = 0$. This fluctuation initiates a self-induced single mode RASER burst in the absence of any RF-pulse. The sech-function Eq.(S18) is symmetric with respect to the time t, so we call this the symmetric Point Spread Function (PSF) which is valid for RASER imaging only if $T_1 = \infty$. An important feature of the spectrum described by Eq.(S19) is that close to the RASER threshold, i.e. $\varepsilon = T_2^*/\tau_{rd} = T_2^*\beta d_0 \approx 1$ the factor $q << 1$ becomes very small, so the associated linewidth w_{sech} of the corresponding spectrum Eq.(S22) is much smaller compared to the linewidth $w = 1/(\pi T_2^*)$ of a Lorentz-shaped peak, the latter representing the standard PSF for SEI. The full width at half maximum w_{sech} is determined by the argument $[\pi T_2^*\omega/2q]$ in Eq.(S19), which for $\varepsilon \approx 1$, $\theta_0 \approx \pi$ and $w = 1/(\pi T_2^*)$ is $w_{sech} = (2/\pi)\ln(2 + 3^{1/2})(\varepsilon - 1)w = 0.84(\varepsilon - 1)w$. For $\varepsilon > 2.2$ the width $w_{sech} > w$, while in the range $1 < \varepsilon < 2.2$ closer to threshold $w_{sech} < w$. For example, at $\varepsilon = 1.4$, $w_{sech} = 0.336\ w = 0.15$ Hz for $T_2^* = 0.7$ s. The argument $w_{sech} < w$ for $1 < \varepsilon < 2.2$ holds even for the case of finite T_1 relaxation associated with an asymmetric PSF, as will be shown in the next section.

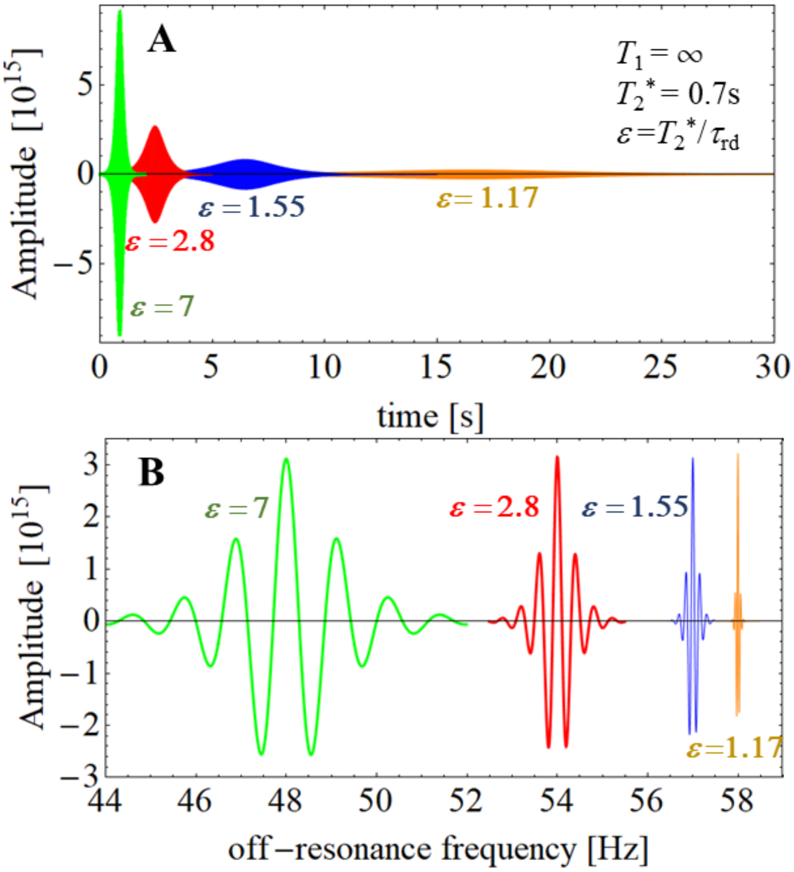

Fig. S2. Numerical simulation of RASER bursts for a single mode ($N = 1$). Simulation parameters: $T_1 = \infty$, $T_2^* = 0.7$ s, $Q = 100$, $V_s = 0.5$ cm^3. Initial values: $\phi(0) = 0$, $A(0) = 10^{13}$, and four different values for $\varepsilon = T_2^*/\tau_{rd} = T_2^*\beta\, d_0$ are assumed. (A) RASER burst versus time for $\varepsilon = 7$ (green), 2.8 (red), 1.55 (blue) and 1.16 (orange). All four simulated signals are symmetrical sech-functions, which are identical to the analytical hyperbolical secant solution given by Eq.(S18) and which are symmetric with respect to the time t_0 (the time where the maximum signal appears). (B) Corresponding simulated phased Fourier-transformed spectra of the four signals shown in Fig. S2(A). The four point spread functions (PSFs) are displaced in the frequency domain (with center frequencies at 48, 54, 57 and 58 Hz) for clarity. Note that in each PSF and with decreasing ε the spectral width and the spacing between subsequent oscillations is decreasing.

Fig. S2(A) shows the numerical simulations of four different PSF for the case $T_1 = \infty$. The transverse spin component A(t) versus time t is plotted for four different parameters $\varepsilon = T_2^*/\tau_{rd} = T_2^*\beta\, d_0$, ranging from ε = 7 (green) far above threshold, ε =2.8 (red), ε =1.55 (blue) to ε =1.16 (orange) close to threshold. The simulation parameters are T_2^* = 0.7s, Q = 100, V_s = 0.5 cm^3 and the initial conditions are $\phi(0) = 0$, $A(0) = 10^{13}$. All four simulated RASER bursts are symmetric sech-functions, which are identical to the analytical solution given by Eq.(S19). Furthermore, each PSF is symmetric with respect to the time of maximum signal, t_0. The time t_0, as given by Eq.(S20), and the width of each burst increases for decreasing values ε. As ε approaches the threshold (ε = 1) the duration or width in the time domain of each PSF becomes arbitrarily long, and the maximum amplitude arbitrarily small. The corresponding Fourier-transformed spectra are shown in Fig. S2(B). The four PSFs are displaced in the frequency space for a better separation. Note that with decreasing ε the spectral width and the period due to the modulation proportional to the factor $\cos(\omega t_0)$ (see Eq.(S19)) in each PSF is decreasing. Close to threshold the corresponding PSFs are much narrower compared to the linewidth of a conventional Lorentz-peak. In our experiments typical measured values are T_1 ~3 s - 5 s and T_2^*= 0.7 s, so the PSF has a width in the range 0.2 Hz < w_{as} < 0.33 Hz and the Lorentzian line width is $w = 1/(\pi T_2^*) = 0.455$ Hz.

3. Case N = 1 including T_1 relaxation: The asymmetric Point Spread Function (a-PSF).

For this next case, we include T_1 relaxation. To our knowledge this has not been discussed in the literature. The dissipation caused by T_1 relaxation, represented by the additional term $-d/T_1$, is introduced in Eq.(S10) for one mode (N = 1). No analytical solution exists for this case, and the symmetry of the PSF given by Eq.(S18) with respect to time t_0 is lost. Fortunately the key properties of the corresponding asymmetric PSF can be evaluated by numerical simulations of Eqs.(S10-S12), including the additional loss term $-d/T_1$.

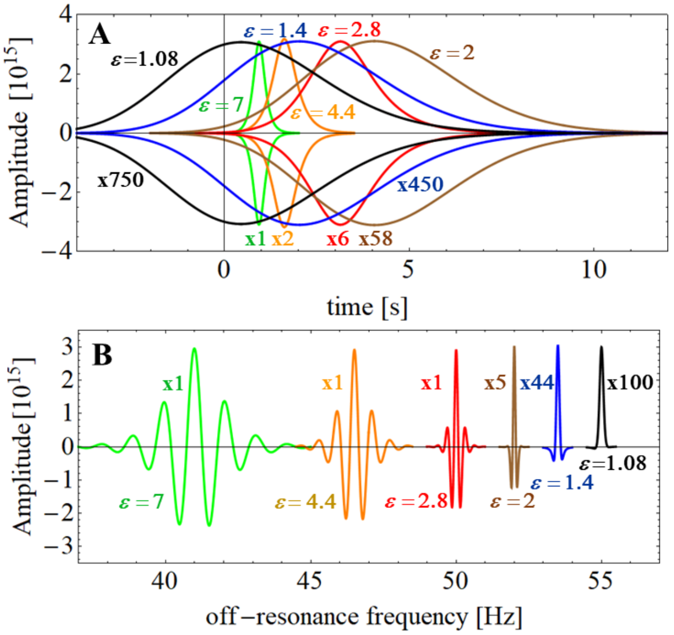

Fig. S3. Series of six simulated asymmetric Point Spread Functions (a-PSF) for T_1 = 6 s. (**A**) Plot of the envelopes of transverse spin components A (RASER bursts) versus time for six different values ε. Simulation parameters: T_1 = 6 s, T_2^* = 0.7 s, Q = 100, V_s = 0.5 cm^3. Initial values: $\phi(0) = 0$, $A(0) = 10^{13}$, and $\varepsilon = T_2^*\beta\, d_0$ = 7 (green), 4.4 (orange), 2.8 (red), 2 (brown), 1.4 (blue) and 1.08 (black). Note the large differences in amplitude and duration of the corresponding a-PSF. (**B**) Corresponding phased Fourier-transformed spectra of the six a-PSFs from Fig. S3(a). Far above threshold, at ε = 7, the peak amplitude including the modulation due to the $\cos(\omega\, t_0)$-term of the spectrum looks quite similar to the spectrum for $T_1 = \infty$ in Fig. S2(b). Close to threshold, at ε = 1.4 and 1.08, the peak amplitude is tens of times smaller compared to ε = 7 and there is nearly no phase modulation visible.

Far away from threshold, at $\varepsilon = 7$, the peak amplitude and the modulation due to the $\cos(\omega t_0)$-term of Eq.(S19) of the spectrum looks quite similar to the spectrum for $T_1 = \infty$ in Fig. S2(B). At $\varepsilon = 1.4$ and 1.08 close to threshold, the peak amplitude is tens of times smaller compared to the peak amplitude at $\varepsilon = 7$. Additionally, close to threshold there is nearly no phase modulation visible in the spectrum, in contrast to the phased spectra close to threshold in Fig. S2(B), which are characterized by a narrow envelope modulated by several oscillations. This difference in the number of periods visible in the PSF in Fig. S2(B) and a-PSF in Fig. S3(B) close to threshold is directly connected to the time of maximum amplitude, t_0. To analyze this key property, the time t_0 as a function of ε has been numerically evaluated for different values of T_1. The result is shown in Fig. S4, where the simulation parameters are given in the caption.

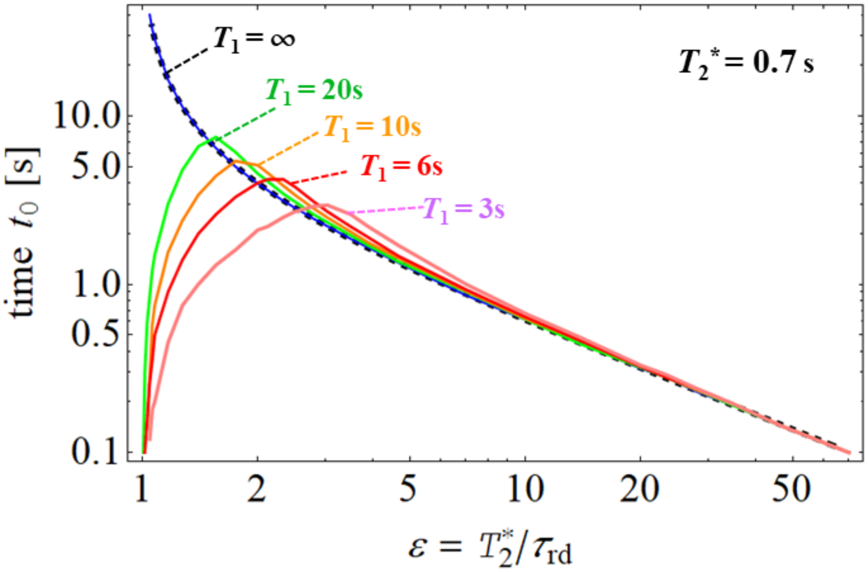

Fig. S4. Time t_0 versus $\varepsilon = T_2^*/\tau_{rd}$ for five different values of T_1. The dotted black and solid blue line correspond to $T_1 = \infty$ and represent the exact expression for t_0 (Eq.(S23)) and the t_0-value evaluated from numerical simulation, respectively. The four other plots represent t_0 derived from numerical simulations for $T_1 = 20$ s (green), 10s (orange), 6 s (red) and 3 s (pink). Simulation parameters are: $T_2^* = 0.7$ s, $Q = 100$, $V_s = 0.5$ cm^3. Initial values: $\phi(0) = 0$, $A(0) = 10^{13}$. For finite T_1 values there is a maximum in the evaluated function $t_0(\varepsilon)$ in the range $1.5 < \varepsilon < 4$. For larger $\varepsilon > 5$ further away from threshold, all curves asymptotically approach the curve for $T_1 = \infty$.

For the case $T_1 = \infty$, t_0 decreases monotonically with increasing ε. Note the dotted curve, which represents the exact solution given by Eq.(S20), is in good agreement with the numerical solution given by the solid blue line. A different behavior is observed for finite values of T_1, here ranging from $T_1 = 20$ s, 10 s, 6 s to 3 s. Starting at $\varepsilon = 1$, t_0 increases with increasing ε until a maximum value is reached in the range $2 < \varepsilon < 4$ and finally $t_0(\varepsilon)$ decreases until approaching the curve for $T_1 = \infty$ in an asymptotic way. Here we state the similarity between the PSF and the a-PSF far from threshold and significant differences close to threshold.

4. Simulation of 1D RASER images for three spin-density profiles

Until now, we have studied PSFs for one single slice ($N = 1$). To describe RASER MRI, the concept of a single PSF is insufficient. Thus, in the following subsections, we will explore the physics of 1D RASER images consisting of many interacting slices ($N >> 1$). Now collective effects dominate the physics of image formation and are essential to describe the image contrast as well as the nonlinear artifacts that arise. To understand these phenomena, we chose simple profiles to reproduce typical RASER MRI features in simulations (see subsections S4 (a-c)).

In all the simulations shown here, we assume parameters matching our experimental conditions: $T_1 = 5$ s, $T_2^* = 0.7$ s (line width w $=1/(\pi T_2^*) = 0.455$ Hz), quality factor $Q = 100$, a cylindrical sample with volume $V_s = 0.5$ cm^3 and diameter $L = 0.8$ cm. All these parameters have either been measured in our ^{1}H RASER-imaging experiments at 166 or at 333 kHz ^{1}H Larmor frequency ($B_0 = 3.9$ or 7.8 mT) with SABRE pumped pyrazine, or correspond to parameters of the setup. The typical range for the magnetic field gradient is $2\cdot10^{-4}$ G/cm $< G_z < 2\cdot10^{-2}$ G/cm, which for a sample dimension of L = 0.8 cm corresponds to image domains in frequency space ranging from 0.67 Hz $< \Delta <$ 67 Hz.

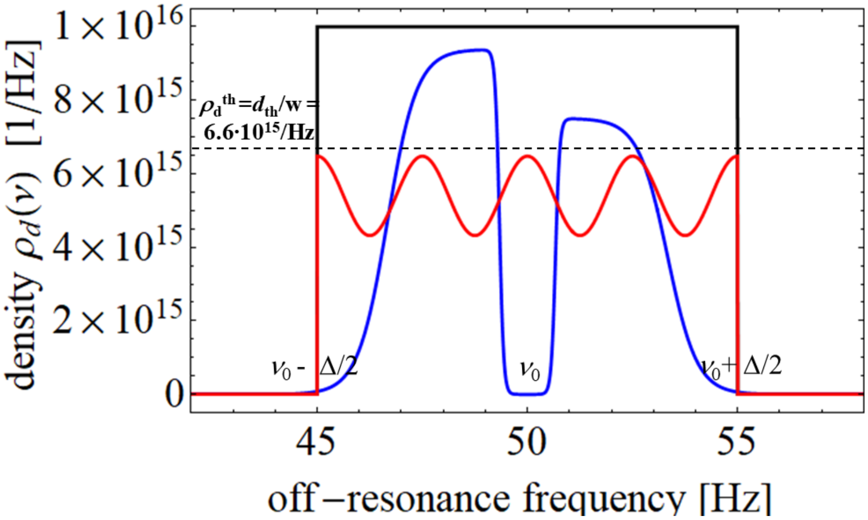

Fig. S5. Three different density profiles $\rho_d(\nu)$ as possible inputs for RASER MRI. All three profiles have an image domain of $\Delta = 10$ Hz centered at the central offset frequency $\nu_0 = 50$ Hz. In the interval 45 Hz $< \nu <$ 55 Hz the three profiles are: A rectangular profile $\rho_d(\nu) = \rho_d^{rect} = 10^{16}$ /Hz, (black), a cosine-modulated profile with offset, $\rho_d(\nu) = 5.5\cdot10^{15}$/Hz $(1 + 0.2 \cos[8\pi(\nu\text{-}\nu_0)/\Delta])$ (red) and two sections with tanh-shaped edges separated by a gap, $\rho_d(\nu) = 4.5\cdot10^{15}$/Hz $(0.5(\tanh[(\nu–\nu_0 + \Delta/3)/0.7] – \tanh[(\nu–\nu_0 + \Delta/15)/0.1]) + 0.4 (\tanh[(\nu–\nu_0 – \Delta/15)/0.1] – \tanh[(\nu–\nu_0 – \Delta/3)/0.7]))$ (blue). The dotted line corresponds to the spin number density per Hz at the RASER threshold $\rho_d^{th} = 6.6\cdot10^{16}$/Hz. Note that all values of the cos modulated profile (red) lie below ρ_d^{th}, which does not mean that no RASER activity is observed.

Three different spin density profiles are defined in Fig. S5 to serve as the input profile for the full model with interaction (Eqs.(S5-S8)): (a) A rectangular profile with a constant value of the population inversion density in the image domain Δ, here $\rho_d(\nu) = \rho_d^{rect} = 10^{16}$/Hz (black). Additionally, a small fluctuation in $\rho_d(\nu)$ is considered in order to understand the contrast mechanism between regions of slightly different population inversion. (b) A cosine-modulated profile with offset, i.e. $\rho_d(\nu) = 5.5\cdot10^{15}$/Hz $(1 + 0.2 \cos[8\pi(\nu–\nu_0)/\Delta])$ (red) to evaluate a possible correction procedure of a distorted image. (c) A profile consisting of two regions (or sections) separated by a 1 mm broad gap, to mimic the experimental conditions. The profile is approximated by tanh-functions, i.e. $\rho_d(\nu) = 4.5\cdot10^{15}$/Hz $(0.5(\tanh[(\nu–\nu_0 + \Delta/3)/0.7] – \tanh[(\nu–\nu_0 + \Delta/15)/0.1]) + 0.4 (\tanh[(\nu–\nu_0 – \Delta/15)/0.1] – \tanh[(\nu – \nu_0 – \Delta/3)/0.7]))$ (blue). With a chosen value for the image domain Δ = 10 Hz and $w_{as} = 0.33$ Hz this corresponds to $N = \Delta/w_{as} = 30$ slices.

The dotted line at ρ_d^{th} = 6.6·10^{15}/Hz indicates the population inversion density at the RASER threshold, which is related to the threshold population inversion d_{th} = ρ_d^{th} w = 3·10^{15}. Note that for the rectangular profile in Fig. S5 all values fulfil $\rho_d(\nu) > \rho_d^{th}$, while for the cosine-modulated profile $\rho_d(\nu) < \rho_d^{th}$. As will be shown in section 4(a), this does not mean that no RASER activity is observed for the cosine-modulated profile.

4(a) Simulation of 1D RASER images for a rectangular profile

The simulation of a RASER image based on a rectangular profile with constant $\rho_d(\nu) = \rho_d^{rect}$ has two different purposes. First, it serves as a simple model system to analyze nonlinear phenomena. Second, it serves as a reference to correct for nonlinear amplitude deformations, which occur in arbitrarily shaped RASER images.

An overall view of how the amplitude and shape of a RASER image depends on different values of the initial population inversion d_0 is shown in Fig. S6. In S6(A), panels I-V, five simulated RASER signals are shown for five different values d_0 = {5.5, 6.6, 10, 15, 30}·10^{16}. As before, the rectangular profile is centered at ν_0 = 50 Hz and with a constant population inversion density $\rho_d(\nu) = d_0/\Delta$ in the frequency range 45 Hz < ν < 55 Hz (Δ =10 Hz). With a given step size $\delta\nu$ = 0.2 Hz this results in N = 50 slices. The green dotted line in Fig. S6(B) indicates the threshold population density $\rho_d^{th} = d_0/\Delta$ = 6.6·10^{15}/Hz.

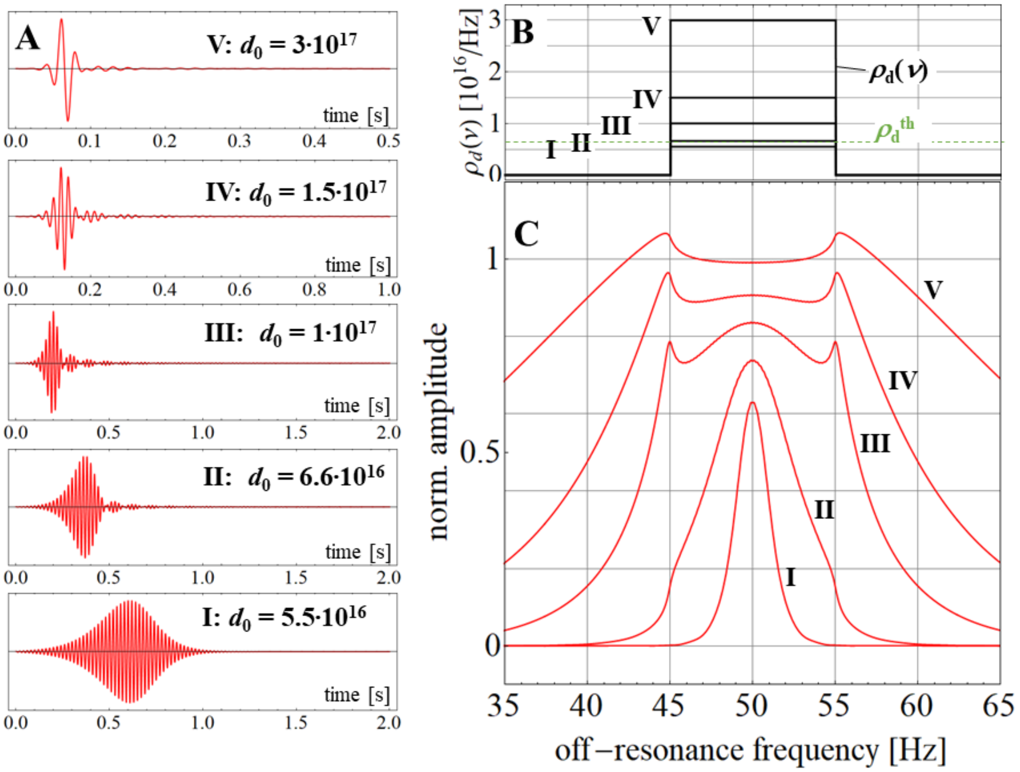

Fig. S6. Simulated RASER images for a rectangular profile $\rho_d(\nu) = d_0/\Delta$. The simulation parameters are: T_2^* = 0.7 s, T_1 = 5 s, Q = 100, V_s = 0.5 cm^3. Number of slices is N = 50, $\delta\nu$ = 0.2 Hz, Δ = 10 Hz, ν_0 = 50 Hz. (**A**) Simulated RASER signals for five different initial population inversions d_0 = {5.5, 6.6, 10, 15, 30}·10^{16} (panels I-V) (**B**) Five rectangular shaped profiles $\rho_d(\nu)$ (I-V, black lines). Green dotted line indicates the threshold population inversion density ρ_d^{th} = 6.6·10^{15}/Hz. (**C**) RASER images obtained after Fourier transformation (absolute mode) from the RASER signals I-V in (A). All images are normalized to the amplitude of image V at ν_0 = 50 Hz. Image I is a narrow peak centered at ν = 50 Hz although $\rho_d(\nu) < \rho_d^{th}$. Images II-V have decaying side lobes outside of the imaging boundaries at ν = 45 Hz and 55 Hz.

Fig. S6(C) shows five RASER MRI images (I-V), obtained after Fourier transformation in absolute mode from five simulated RASER signals in S6(A). Let us start with image I in S6(C) ($d_0 = 5.5\cdot10^{16}$), where $\rho_d(\nu) < \rho_d^{th}$. This means that the population inversion within a slice of a width w, ($\rho_d(\nu)\cdot$w) is smaller than the threshold population inversion d_{th} required to start RASER activity. Therefore, at first glance no RASER action is expected for all $N = 50$ slices. Surprisingly, a RASER burst is visible (burst I in S6(A)) and the corresponding image I in S6(C)) is a symmetrical peak with a maximum at 50 Hz and a width much narrower than Δ. The reason for this shape is the cooperative action between all 50 slices. The slices close to the center at 50 Hz successfully surpass the threshold through the cooperation with their neighbors.

In contrast to that, the slices on the edges (at 45 and 55 Hz) are strongly suppressed and the simulated image amplitude is maximal in the center of the image domain.

The next case for $d_0 = 6.6\cdot10^{16}$ is shown in image II in S6(C), where $\rho_d(\nu) = \rho_d^{th}$ holds. Now, all slices in the image domain Δ are RASER active and contribute to the image. Nonetheless, the image does not have a rectangular shape, but a bell-shaped image with a maximum amplitude in the center is formed. Furthermore, decaying sidelobes outside from the image boundaries arise. We found that cooperative action between all slices leads to broad and complicated spectra of each slice and are responsible for the signal outside the image domain Δ (see Fig. S7).

In image III ($d_0 = 1\cdot10^{17}$) the amplitude at the edges is nearly as high as the amplitude in the center and the decaying sidelobes are more pronounced. In this case, the slices at the edges of the image domain benefit from the enhanced cooperative interaction between all slices. This effect is even more pronounced in the images IV ($d_0 = 1.5\cdot10^{17}$) and V ($d_0 = 3\cdot10^{17}$), with $\rho_d(\nu) >> \rho_d^{th}$, where the amplitude in the center is comparable or smaller in size with respect to the amplitude at the image boundaries.

Next, we study the sensitivity of a RASER image with respect to small disturbances in the polarization distribution in the profile $\rho_d(\nu)$.To this end, we compare simulated RASER images with standard imaging based on Lorentz shaped PSFs. As input profile, we assume a rectangular input profile $\rho_d(\nu)$ for $d_0 = 9\cdot10^{16}$ and for an image domain ranging from 45 Hz to 55 Hz (Δ = 10 Hz, $\delta\nu$ = 0.151 Hz, N = 66). On this rectangular profile, a small perturbation is added in the form of a rectangular shaped hole in the center ν_0 = 50 Hz. The width of this hole is one half of the natural linewidth, i.e. w/2 = $1/(2\pi T_2^*)$ = 0.23 Hz, and the amplitude is 20% smaller compared to the maximum of $\rho_d(\nu)$. In this case, the smallest value in the hole is still above the threshold population density $\rho_d^{th} = 6.6\cdot10^{15}$/Hz and therefore all N = 66 slices are RASER active. The resulting simulated RASER image is depicted in Fig. S7(B) as I (black).

For comparison, an image based on a superposition of Lorentzian PSFs is simulated assuming a Lorentzian PSF of width w, i.e. $L(\nu,\nu^*) = 1/[1 + (\nu - \nu^*)^2/\mathrm{w}^2]$. This PSF is folded with the profile $\rho_d(\nu)$ shown in Fig.7 (A) to obtain the image $S_\mathrm{L}(\nu) = \int_{-\infty}^{\infty} L(\nu,\nu^*)\,\rho_\mathrm{d}(\nu^*)\,d\,\nu^*$ depicted in Fig S7(B) as I (red).

Comparing both images in Fig. S7(B), there are two apparent differences in the RASER image: First, similar artifacts as shown in panel III of Fig. S6(C) for a similar population inversion arise. The amplitude of the RASER image with respect to $\rho_d(\nu)$ inside the imaging domain is deformed and sidelobes outside of the image domain are more pronounced. Both of these artifacts are typical for a rectangular input profile above the threshold density and are not attributed to the introduced small perturbation. Secondly, in the RASER image there is a minimum in intensity in the center of the image at ν_0 = 50 Hz. The amplitude of this perturbation is about three times higher and the slope at the edges three times steeper. This simple example demonstrates the advantage and problems of RASER imaging, which is sensitive to small perturbations, but suffers from amplitude deformations inside of Δ and side lobes outside of Δ.

Spectra of seven representative slices are chosen for Fig. S7(C). Each of these spectra is obtained after Fourier transformation of the respective RASER signal of slice μ. The slices at the image boundaries (μ = 1 and 66) are depicted in brown, slice μ = 33 at the center is depicted in blue, while the slices between these extremes are shown in orange (μ = 12 and 54) and green (μ = 24 and 42). The peak amplitude of the spectrum in the center (μ = 33, blue) is significantly smaller compared to slices μ = 24 and 42 (green). This motivates the increased sensitivity of RASER imaging with respect to small changes in the amplitude of $\rho_d(\nu)$. The width of each spectrum in Fig. S7(C) is close to w. Furthermore, all spectra feature broad sidelobes left and right of the peak maxima, which can extend out of the image domain Δ. The highly resolved local hole with w/2 width cannot be explained by the width w for the individual N = 66 slices. Instead, the collective interaction between all slices generates the local image contrast. This interaction involves collective phenomena such as synchronism, line collapse and other non-linear phenomena which enhance local variations $\rho_d(\nu)$ in a non-linear way.

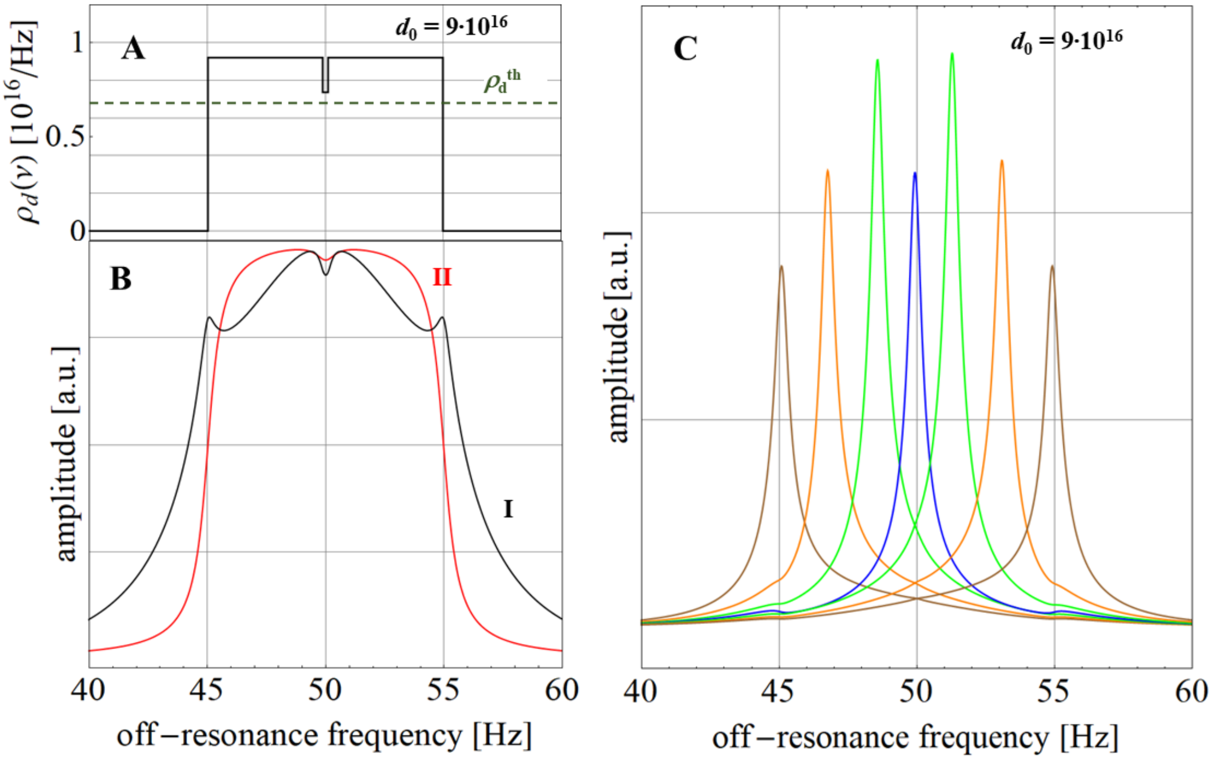

Fig. S7. Comparison between simulated RASER images and imaging based on Lorentzian shaped PSFs. (A) A rectangular input profile $\rho_d(\nu)$ is assumed with Δ = 10 Hz and $d_0 = 9\cdot10^{16}$ with a small rectangular hole in the center at ν_0 = 50 Hz. The width of this hole is w/2 = 0.23 Hz and the depth is 20% with respect to the maximum of $\rho_d(\nu)$ and still above the threshold population density (dashed green line; $\rho_d^{th} = 6.6\cdot10^{15}$/Hz). (**B**): Simulated RASER image (I, black) and image based on a superposition of Lorentzian PSFs (II, red) of the profile $\rho_d(\nu)$ shown in (A). **(C)** Seven representative spectra of the 66 slices: μ = 1 and 66 (brown), 12 and 54 (orange), 24 and 42 (green) and μ = 33 (blue) obtained after Fourier transformation in the absolute mode. Note the broad shoulders left and right from the peak maxima in each of the spectra, which arise from the collective interaction between all slices. Simulation parameters for the RASER image: N = 66 slices, $\delta\nu$ = 0.151 Hz, T_2^* = 0.7s, T_1 = 5 s, Q = 100. The width of the Lorentzian PSFs is w = $1/(\pi T_2^*)$ = 0.455 Hz.

4(b) Simulation of 1D RASER images with a cosine-modulated profile

In the next step we will study simulations of RASER images with more complicated profiles. One interesting example is shown in Fig. S8, depicting simulations of the time dependent RASER signals (A,D,G) and the corresponding RASER images (B,E,H) for a given offset superimposed by a cosine-modulated profile (red lines in insets of (A,D,G)).

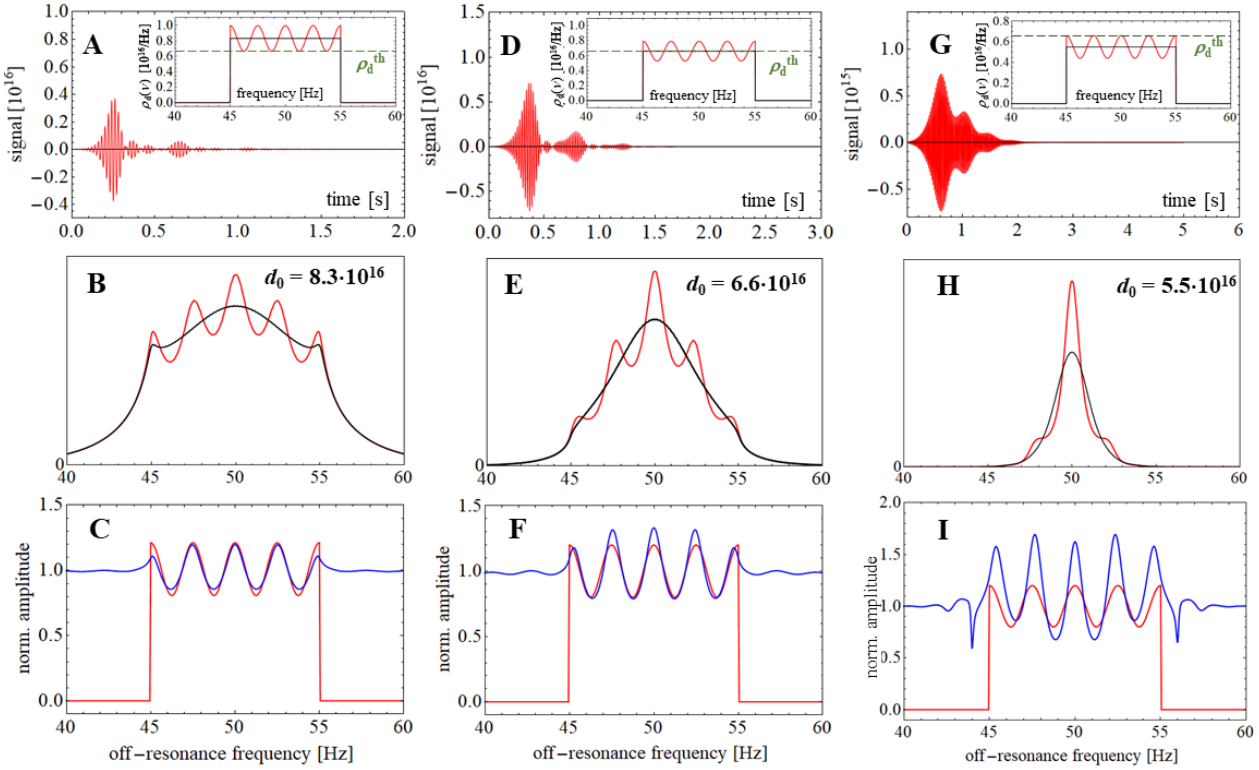

Fig. S8. Simulated RASER images for a cosine-modulated profile with offset. (**A**,**D**,**G**): RASER signal versus time simulated for $N = 50$ slices (Δ = 10 Hz, $\delta\nu$ = 0.2 Hz, ν_0 = 50 Hz, T_1 = 5 s, T_2^* = 0.7 s) and for three different offsets of the cosine-modulated profiles (insets, red), $\rho_d(\nu) = C_0(1 + 0.2\cos[8\pi(\nu-\nu_0)/\Delta])$, where $C_0 = d_0/\Delta$ = $8.3\cdot10^{15}$/Hz (A), $6.6\cdot10^{15}$/Hz (D) and $5.5\cdot10^{15}$/Hz (G). The three profiles in (A,D,G) correspond to an initial population inversion of $d_0 = 8.3\cdot10^{16}$, $6.6\cdot10^{16}$, and $5.5\cdot10^{16}$, respectively. A black, rectangular profile in the insets of (A,D,G) serves as a reference for a global rectangular correction. The dotted green line is the density at threshold ρ_d^{th} = $6.6\cdot10^{15}$/Hz. (**B**,**E**,**H**) represent the simulated RASER images for the cosine-modulated (red) and the rectangular (black) profiles for the insets of (A,D,G), respectively. (**C**,**F**,**I**): Corrected RASER images are obtained through division of the amplitudes between the red and black curves in (B,E,H), respectively. Red lines correspond to the normalized spin density profiles $\rho_d^*(\nu) = \rho_d(\nu)/C_0$. The corrected image in (C) ($\rho_d(\nu) \geq \rho_d^{th}$) is in good agreement with the normalized profile $\rho_d^*(\nu)$. In (F,I), $\rho_d(\nu) \leq \rho_d^{th}$, the corrected image deviates from the normalized $\rho_d^*(\nu)$.

The simulation parameters and boundary conditions are given in section 5 and in the caption of Fig. S8. The threshold population density, $\rho_d^{th} = 6.6\cdot10^{15}$/Hz is indicated as green dashed line in the insets of Fig. S8(A,D,G). In order to resolve all the details, N = 50 slices are assumed for the simulations, which corresponds to an image domain of $\Delta = N \cdot \delta\nu$ = 10 Hz. The four periods of the profile have a modulation depth of 20%, i.e. $\rho_d(\nu) = C_0(1 + 0.2\cos[8\pi(\nu-\nu_0)/\Delta])$, where the factor $C_0 = d_0/\Delta$ quantifies the average spin density. Three different offset values of C_0 are chosen, as indicated in the insets (A,D,G). In the first case, Fig. S8(A), $C_0 = 8.6\cdot10^{15}$/Hz, so $\rho_d(\nu) \geq \rho_d^{th}$ holds over the whole image domain. The Fourier-transformed image in the absolute mode in S8(B), red line, reflects the four periods of the profile, and the rough envelope of the image with a maximum amplitude in the center at ν_0 = 50 Hz is deformed relative to the profile. We know already from the previous section that the image of a rectangular profile is deformed and characterized by a Gaussian-like shape, reaching maximum amplitude at the center.

A correction of the deformed image of the cosine-modulated profile is possible by using the corresponding image of a rectangular profile. We call this procedure a global rectangular correction. The rectangular profiles are drawn as black lines in the insets of Figs. S8(A,D,G), and the constant value corresponds to the average value of the cosine-modulated profile, i.e. $\rho_d^{rect}(\nu) = C_0$. The black lines in (B,E,H) represent the reference images based on the corresponding three rectangular profiles in the insets of (A,D,G). The global rectangular correction procedure consists of dividing the amplitude of the cosine-modulated image by the amplitude of the reference image for each frequency. The results of this procedure are shown as blue lines in Figs. S8(C,F,I). The images of the cosine-modulated profile corrected in this manner can be directly compared to the normalized profiles (red lines in (C,F,I)), the latter being defined by $\rho_d^*(\nu) = \rho_d(\nu)/C_0$. In Fig. S8(C) there is good agreement between the corrected image (blue line) and the normalized profile (red line) in the image domain 45 Hz < ν < 55 Hz.

A case where half of the population inversion densities $\rho_d(\nu)$ are below the threshold density ρ_d^{th} is shown in Figs. S8(D,E,F). The corrected image in (F) is not perfectly reflecting the normalized profile: The maxima of the corrected image are about 20% larger. An extreme case is demonstrated in Figs. S8(G,H,I), where all slices individually are below the RASER threshold $\rho_d(\nu) < \rho_d^{th}$. One might think that RASER activity is impossible. This would be a misconception however, due to the fact that all slices are RASER active due to the cooperative-nonlinear interaction. The corrected image in Fig.S8(I) retains roughly the shape of the normalized profile, but at the cost of a large deformation in the amplitude, with the minima of the corrected image being much closer to the normalized profile than the maxima.

We found that in general for small modulation depths or variations close above threshold, the images reflect the normalized profile. At higher modulation depths and further away from threshold, the nonlinear amplitude deformations increase significantly. For example, for a cosine-modulated profile with 50% modulation depth and $\rho_d(\nu) < \rho_d^{th}$, the image transforms into one dominant peak at the center and very small peaks close to the local maxima of $\rho_d(\nu)$. One open question for RASER MRI applications is whether there is an algorithm capable of correcting these nonlinear deformations.

4(c) Simulation of two sectors with tanh shaped edges separated by a gap

In the last example we attempt to come closer to our real experiment, in which two sections of a cylindrical sample with 8 mm inner diameter are separated by a gap of 1 mm size and pumped separately by SABRE. We determined the 1D projection experimentally with hyperpolarized high-resolution SEI (see Fig.5(A), main text). These 1D images can be approximated using two step-like functions separated by the gap and the rising and falling edges of each step by tanh-functions. Examples of normalized two-chamber profiles are sketched in the insets of Figs. S10(A). All five profiles correspond to an analytical expression given by the sum over four tanh step functions, i.e. $\rho_d^*(\nu) = A_1(\tanh[(\nu - \nu_0 + b_1)/w_1] - \tanh[(\nu - \nu_0 + b_2)/w_2]) + A_2(\tanh[(\nu - \nu_0 - b_3)/w_3] - \tanh[(\nu - \nu_0 - b_4)/w_4])$. The constants A_1 and A_2 define the maximum amplitudes of both steps, ν_0 is the frequency offset, b_1, b_3 (b_2, b_4) denote the two positions of the rising (falling) edges of the tanh step functions relative to ν_0, and w_1, w_3 (w_2, w_4) define the widths associated to the rising (falling) edges. The normalization of $\rho_d^*(\nu)$ to the value one is hereby related to the larger value of A_1 or A_2, respectively. The amplitudes of A_1 or A_2 are not necessarily equal, but depend on the pumping conditions and T_1 relaxation rate of each of the two chambers. The maximum value of the profile $\rho_d^*(\nu)$ can be compared to the normalized population inversion density at threshold, i.e. with $\rho_d^{*th} = \Delta\, \rho_d^{th}/d_0$.

Let us first analyze one experimental result from Fig. 5(B), main text. At Δt = 8 s, the population inversion d_0 has decayed significantly and all slices are below the RASER threshold. The corresponding RASER signal is shown in Fig. S9(A), while S9(B) depicts the phased spectrum and S9(C) the absolute spectrum identical with Fig. 5(B), Δt = 8 s. The applied gradient G_z = 5.78 mG/cm spans an image domain of $\Delta = \gamma_H G_z L$ = 19.5 Hz. The signal in S9(A) is noisy and symmetrical with respect to the maximum at t_{max} = 1.5 s. The Fourier transformed spectrum in the phased mode (S9(B)) has the shape of a phase modulated sech function, as discussed in section 3. The corresponding absolute phased spectrum in S9(C) is a symmetrical

peak centered at 127.7 Hz and the full width at half maximum (FWHM) is 0.6 Hz. This width is broader compared to the Lorentzian linewidth w = 0.45 Hz. Taking into account the result for the line shape and width for single slices in Fig. S7(C) this indicates that only a few cooperating coupled slices are responsible for the shape and width of the observed spectrum.

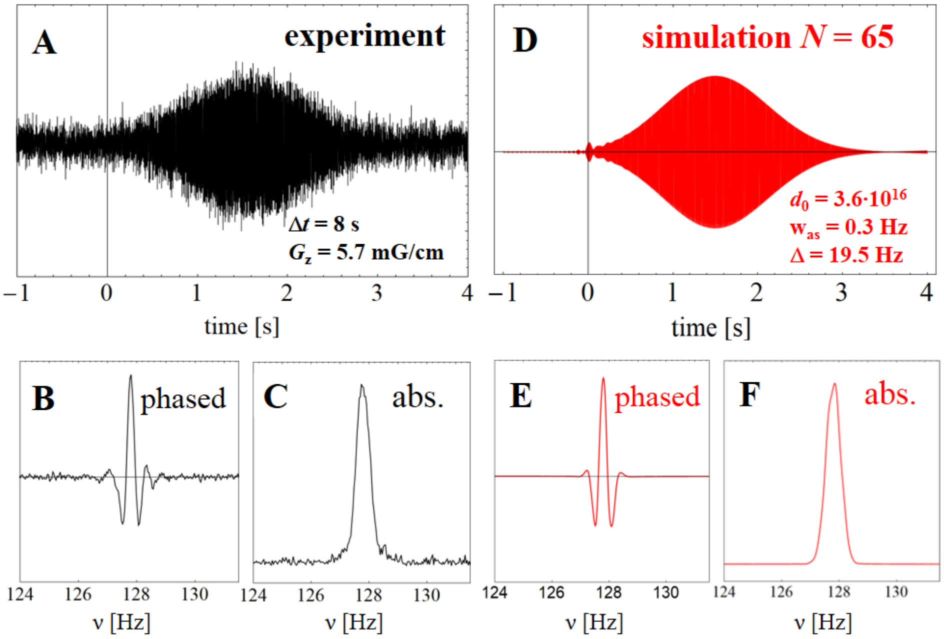

Fig. S9. Measured (A,B,C) and simulated (D,E,F) RASER burst for an image at $\rho_d(\nu)$ below threshold. The experiment shown in (A,B,C) is identical to Fig. 5(B), Δt = 8 s from the main text. (**A**) Measured RASER signal acquired at B_0 = 7.8 mT (333 kHz ^{1}H) and G_z = 5.7 mG/cm with corresponding Fourier transformed spectrum in the phased (B) and in absolute mode (C). The line width at FWHM in (C) is 0.6 Hz. (**D**) Simulated RASER burst (N = 65, d_0 = 3.6·10^{16}, Δ = 19.5 Hz, ν_0 = 132.7 Hz, $\delta\nu$ = 0.3 Hz, T_1 = 5 s, T_2^* = 0.7 s) and corresponding spectrum in the phased (E) and absolute mode (F). Note the large noise contribution in A, which indicates that RASER action is measured close to the threshold density ρ_d^{th} = 6.6·10^{15}/Hz. All simulated signals and images based on Eqs.(1-4) in (D,E,F) are in good agreement with the measurements in (A,B,C), if a profile $\rho_d(\nu) = C_0\{0.5(\tanh[(\nu - \nu_0 + 6.5)/1.4] - \tanh[(\nu - \nu_0 + 3.6)/1.4]) + 0.25(\tanh[(\nu - \nu_0 - 3.6)/1.4] - \tanh[(\nu - \nu_0 - 6.5)/1.4])\}$ is assumed, where C_0 = 6.5·10^{15}/Hz and ν_0 = 132.7 Hz. Although $\rho_d(\nu) < \rho_d^{th}$, the interaction between all 65 coupled slices enables RASER action and mainly the center of the image domain is observed. This leads to RASER action associated to a corresponding symmetric narrow image, which has a width of 0.6 Hz > $\delta\nu$ = 0.3 Hz and a maximum value at 127.7 Hz.

In fact, the observed features in Figs. S9(A,B,C) can be simulated based on the theory (Eqs. S5-S8) using an equidistant slicing of the image domain with $\delta\nu$ = 0.3 Hz. Considering the experimental image domain of Δ = 19.7 Hz, the simulations require N = 65 coupled slices. For Δt = 8 s the polarization on the right half has decayed faster compared to the left half (A_2 = 0.25 < A_1 = 0.5) and the relaxation rate on the sample walls is larger than in the bulk. Thus, the initial spin density profile is assumed as two peaks, which are each narrower than Δ/2, i.e. $\rho_d(\nu)$ = 6.5·10^{15}/Hz $\{0.5(\tanh[(\nu - \nu_0 + 6.5)/1.4] - \tanh[(\nu - \nu_0 + 3.6)/1.4]) + 0.25(\tanh[(\nu - \nu_0 - 3.6)/1.4] - \tanh[(\nu - \nu_0 - 6.5)/1.4])\}$ (see Fig. S10(A), panel I).

The simulated RASER burst based on the chosen $\rho_d(\nu)$ is nearly symmetrical with respect to its maximum amplitude at t_{max} = 1.5 s (see S8(D)). Both this signal and the phased and absolute spectra in S8(E,F) are in good agreement with their experimental counterpart. Note that the absolute spectrum in Fig. S9(F) is identical with Fig. 4(C), panel I.

Further away from the threshold, more slices are involved in the image and consequently the nonlinear effects are more prominent. Fig. S10(A) shows the simulated RASER signals versus time for five different

values of the initial population inversion $d_0 = 3.6\cdot10^{16}$ (I), $6.3\cdot10^{16}$ (II), $1.2\cdot10^{17}$ (III), $1.5\cdot10^{17}$ (IV) and $2\cdot10^{17}$ (V). Further simulation parameters are given in the caption of Fig. S10. The duration of the RASER signal becomes longer with decreasing d_0. The corresponding normalized spin density profiles $\rho_d^*(\nu)$ (red lines) are depicted in the insets. For clarity normalized $\rho_d^*(\nu)$ are used here. The green dashed lines indicate five different values for the normalized threshold population inversion densities ρ_d^{*th}. In S10(A) panel I $\rho_d^{*th} = 1.06 > \rho_d^*(\nu)$ is assumed in the image domain $\Delta = 19.7$ Hz, thus a narrow image of the left half is expected. In S10(A) panel V, $\rho_d^{*th} = 0.4 < \rho_d^*(\nu)$ holds, so large nonlinear effects are expected. The five profiles $\rho_d^*(\nu)$ in the insets of S10(A) differ in shape and amplitude ratio between the left and right half of the phantom, which takes into account two different overall T_1 relaxation rates ($T_1 = 5$ s, 3 s for the left and right half, respectively) as well as locally augmented relaxation rates at the walls of the sample chambers.

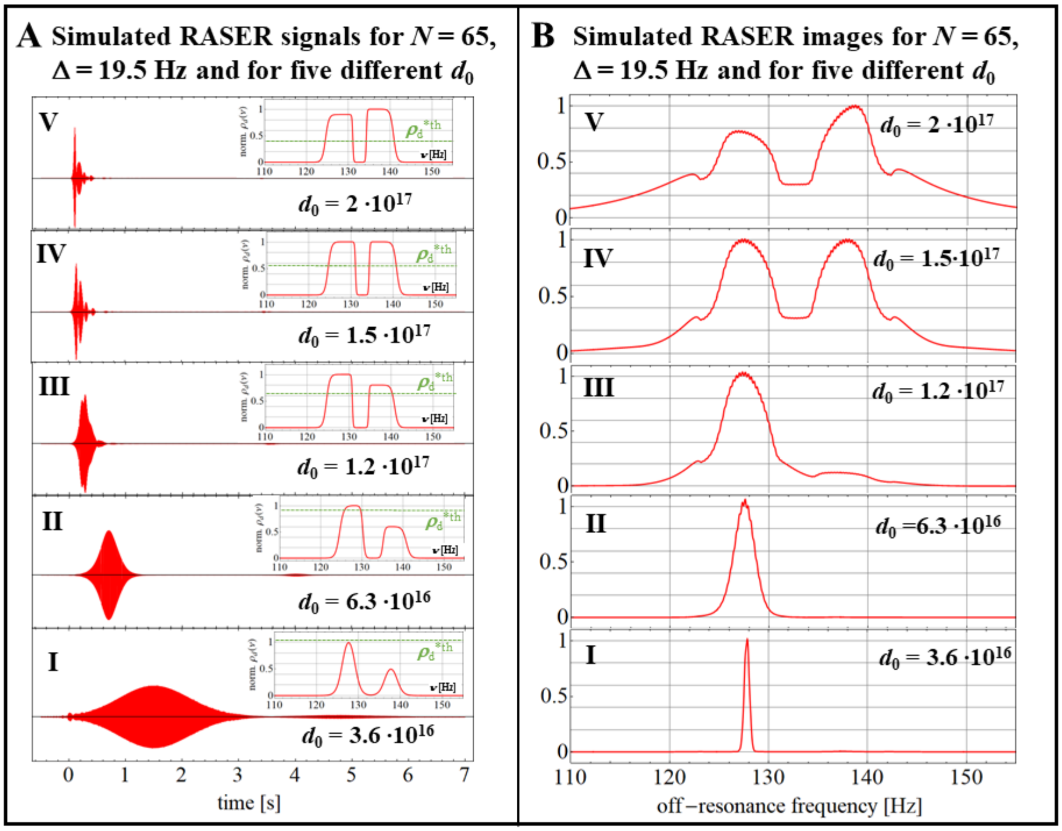

Fig. S10. Simulation of RASER images for different $\rho_d(\nu)$ and d_0 for two chambers separated by a gap of Δz = 1 mm. (A). RASER burst signals at the different $\rho_d(\nu)$ and d_0. Simulation parameters: $N = 65$, $\Delta = 19.5$ Hz, $\delta\nu = 0.3$ Hz, $\nu_0 = 132.7$ Hz, $T_1 = 5$ s, $T_2^* = 0.7$ s. Insets: Corresponding normalized spin density profiles $\rho_d^*(\nu)$ (red lines). Green dashed lines indicate five values for the normalized threshold population inversion densities, i.e. $\rho_d^{*th} = 1.06$ (I), 0.94 (II), 0.61 (III), 0.56 (IV), and 0.4 (V). (**B**) The five RASER spectra I - V represent the corresponding images, obtained from the RASER signals in (A) following Fourier transformation in the absolute mode. The images reflect roughly the features of the experimentally measured RASER images (Fig. 5B, main text), including the side lobes in images IV,V appearing outside the image boundaries at 122.95 Hz and 142.45 Hz. For convenience the normalized population inversion density $\rho_d^*(\nu)$ is introduced in the insets of Fig. S10(A), where the maximum of the profile $\rho_d^*(\nu)$ is normalized to unity. The corresponding normalized threshold population density ρ_d^{*th} is indicated by the dotted green lines.

The five RASER spectra I-V in Fig. S10(B) represent the corresponding images, which are obtained from the RASER signals in S10(A) after Fourier transformation in the absolute mode. With increasing d_0, the images change from (I) a narrow peak, to (II) a broader image centered at 127.7 Hz, (III) an image where the right half starts to be visible, (IV) an image where both halves with equal amplitudes separated by the gap appear and finally (V) to an image where the right half is larger in amplitude. The simulated image I in

S10(B) is identical to Fig. S9(F) and is discussed above. The other four simulated images II-V roughly reflect the features of the experimentally measured RASER images in Fig. 5B, main text, including the side lobes in images IV,V appearing outside the image boundaries and non-zero values in the gap.

There are two additional phenomena, sometimes observed in the measured images: Pronounced ripples and regions with strongly changing amplitudes. For example, ripples can be seen in Fig. 5(B), main text, at Δt = 1 s, 4 s and 5 s at positions 1 mm < z < 2 mm. An example for peaks with strongly changing amplitudes is shown in Fig. 5(B), main text, for Δt = 1 s at position z = –1 and +3 mm. Possible imaging artifacts for RASER MRI are discussed in the next section.

5. 2D RASER image artifacts and 1D projections

This section focuses on the artifacts that can arise in 1D and 2D RASER images. The manuscript mainly discusses 1D images, also called projections, but in Fig. 4(A) and (B) of the main text two different 2D images are shown, a spin echo image for reference and a RASER image. These 2D images are generated using projection reconstruction of 30 1D images measured from 30 angular directions. In these 2D images, artifacts arise due to the projection reconstruction algorithm as well as within the 1D projections themselves.

Projection reconstruction generates star artifacts. These are well known, as projection reconstruction is widely used e.g. in imaging methods such as computed tomography (CT). The star artifacts are more pronounced further away from the center. This can for example be seen in the bottom left corner of Fig. S11(A) and the top left and right corner of Fig. S11(B). Star artifacts can be reduced and the angular resolution increased by measuring more projections.

Other artifacts and features in the 2D images stem from the p-H_2 delivery system, necessary for SABRE pumping. The p-H_2 is introduced through a capillary in each of the chambers. In the SEI, they can be seen as dark spots, each corresponding to the location of a capillary for p-H_2 supply. For RASER MRI, the capillaries can additionally result in nonlinear distortions of a given projection, as RASER MRI is very sensitive to local fluctuations in polarization. Additionally, the p-H_2 bubbling introduces a motion of the liquid in both chambers. This motion stops after about 1-2 s. To ensure a motion-free reference image, Δt = 5 s is chosen for SEI. The RASER image however, is very sensitive to the initial population inversion as visualized in Fig. 5 of the main text. To ensure that both chambers have a population inversion in a regime where an image is formed, Δt = 2 s is chosen for RASER MRI. This leaves the RASER image more susceptible to slight residual motion.

Most artifacts can be identified in the 1D projections. Thus, for the 2D RASER image in Fig. 4(B) of the main text, five projections (I-V) are selected. Their gradient directions are drawn as colored lines in Fig. S11(B). The 1D images of these chosen angles are depicted in Fig. S11(C). When choosing a gradient direction perpendicular to the gap between the half circles of the phantom, the gap can be identified as a minimum amplitude in the middle of the projection. Here, projections III (green) and IV (blue) are close to this condition. For the 1D images in Fig. 3 of the main text, the gradient is chosen perpendicular to the gap as discussed there. A gradient parallel to the direction of the gap yields a projection without a minimum amplitude in the middle, similar to the projection of a circle (see Fig. S11(C), projection V, orange).

The most prominent artifacts in the 2D RASER image are interference lines through the entire image. They can be identified within the 1D projections that have a gradient direction perpendicular to the observed interference line. In the projection they can be identified as “spikes” at the given position. This is visualized exemplarily for two interference lines, marked by stars and arrows in the 2D image. They stem from projections II (red) and III (green), respectively. These artifacts can also be identified in the projections and are encircled and marked with stars in Fig. S11(C). One possible reason for such interference artifacts is

the sensitivity of the coupled RASER modes to local disturbances. Disturbances can be caused by residual motion as described above, the capillaries for p-H_2 delivery and fluctuations in the profile $\rho_d(\nu)$, produced by the SABRE pumping.

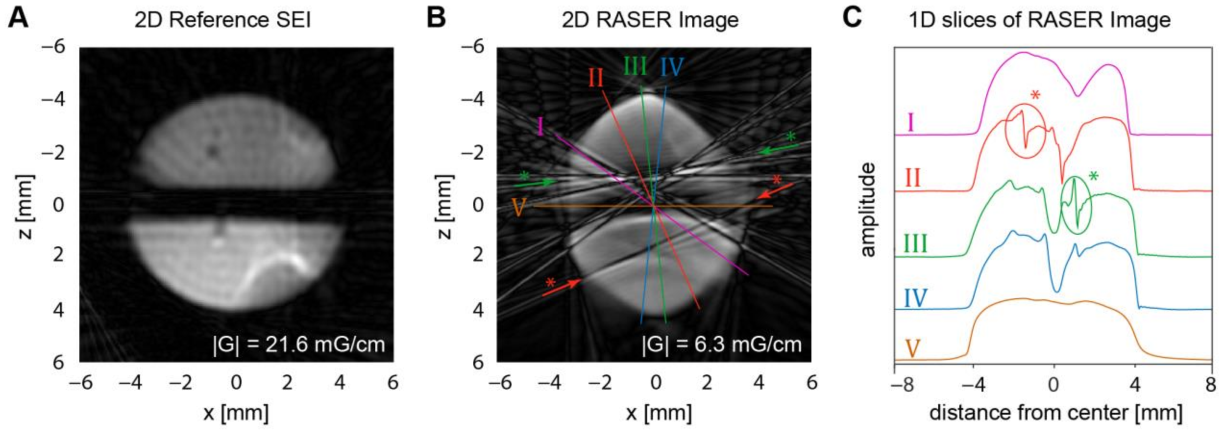

Fig. S11. 2D SEI and 2D RASER image from the main text (Fig. 4), as well as selected RASER imaging projections. (**A**) 2D SEI, (**B**) 2D RASER MRI and (**C**) selected RASER image projections measured at 3.9 mT. To obtain (**A**) and (**B**), 30 projections are measured with the sequence in Fig.2(C, D), each from different angles by varying G_x and G_z such that $G_x^2+G_z^2$ = const. The 2D images are obtained after projection reconstruction. In (**A**), the two capillaries used for p-H_2 supply are visible around x = –1 mm, z = 0.5 mm and x = –1.5 mm, z = –2 mm for each chamber. In (**C**), five 1D projections (I-V) used to reconstruct the 2D RASER image are displayed. The direction of the gradient for each of the five projections is depicted as a colored line (I-V) in (**B**). The RASER image (**B**) is recorded at a 3.5 times smaller gradient than (**A**), but both spatial resolutions are similar. The RASER image is plagued by interference lines. Two of these lines are marked in both (**B**) and (**C**) by stars corresponding to projection II (red) and III (green), respectively. The direction of the interference line in the 2D RASER image in (**B**) is perpendicular to the gradient direction (marked by arrows). These two artifacts in (**B**) can be identified as spikes in the corresponding 1D projections (II) and (III) in (**C**) and are highlighted by a star. The origin of these artifacts is discussed in the text.

Further artifacts that arise in a 1D RASER image are leaking signal into a gap as well as sidelobes that arise outside of the image domain. They are small in the 2D images depicted here, but can play a major role in 1D RASER images recorded at other experimental conditions. These leaking and sidelobe artifacts are discussed extensively in section S4a for a rectangular profile $\rho_d(\nu)$.

Other phenomena such as global and relative dipolar shifts might contribute to the observed artefacts. Past studies showed that global dipolar shift effects are quite small for SABRE pumped samples. For example, the total ^{1}H dipolar shift generated by the SABRE pumped liquid, as described in the supplement of (*19*), was in the order of 200 mHz (B_{dip} ~5 nT). This global dipolar field decays with time as the population inversion d_0 is depleted during the RASER burst. This time-dependent global dipolar field may alter the shape of the image by up to 0.2 Hz, which is on the order of the width of one slice $\delta\nu = w_{as}$. Whether these time-dependent dipolar shifts contribute to the interference artifacts or could produce chaotic regions is still an open question. To keep the RASER MRI model as simple as possible, we also neglected dipolar contributions in the simulations.

6. Three invariance principles for RASER MRI

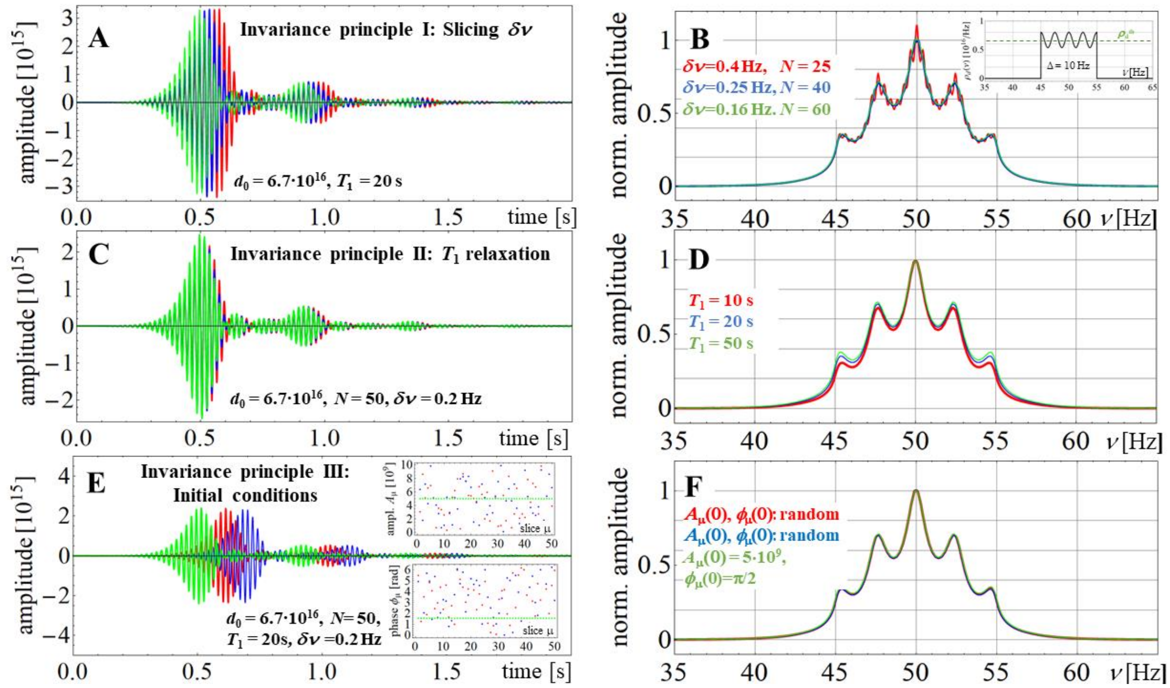

Fig. S12. Three invariance principles for RASER images for a sinusoidal input profile. *Invariance principle I*: Different slicing values $\delta\nu$. (**A**) Simulated RASER signals and (**B**) Fourier transformed signal for $\delta\nu$ = 0.4 Hz (N = 25, red), $\delta\nu$ = 0.25 Hz (N = 40, blue), and $\delta\nu$ = 0.166 Hz (N = 60, green). *Invariance principle II*: Different T_1 values. (**C**) Simulated RASER signals and (**D**) Fourier transformed signals for three different T_1 relaxation times: T_1 = 10 s (red), 20 s (blue) and 50 s (green). *Invariance principle III*: Different initial conditions $A_\mu(0)$ and $\phi_\mu(0)$. (**E**) Simulated RASER signals and (**F**) Fourier transformed signals for one fixed amplitude and phase with $A_{\square}(0) = 5\cdot10^9$, $\phi_\mu(0) = \pi/2$ (green), and two different random initial conditions $A_\mu(0) = 10^{10}\cdot$ Random[] and $\phi_\mu(0) = 2\pi\cdot$ Random[] (red and blue). The insets in (**E**) displays the initial conditions $A_\mu(0)$ and $\phi_\mu(0)$ for each of the N = 50 slices. The function Random[] produces a random number in the interval [0,1]. All Fourier transformed signals are displayed in the absolute mode. The sinusoidal input profile used in all simulations (A-F) is depicted in the inset of (**B**) with $\rho_d(\nu) = 6.6 = 10^{15}$/Hz $(1 + 0.2\cos[8\pi(\nu\text{-}\nu_0)/\Delta])$, where ν_0 = 50 Hz and Δ = 10 Hz. The green dotted line in the inset is the threshold population density $\rho_d^{th} = 6.6\cdot10^{15}$/Hz. Simulation parameters: $d_0 = 6.7\cdot10^{16}$, Q = 100, T_2^* = 0.7 s, V_s = 0.5 cm^3. The other parameters N, T_1, and $\delta\nu$ are indicated in Figs.(A-F).

This chapter focuses on simulations of the invariance principles introduced in the main text and in section 1 of the supplementary material. Invariance principles allow to reduce complexity (with associated conservation laws) and to unravel the basic features of a given physical theory. Given that $T_1 >> T_2^*$, loss effects in the total image amplitude can be neglected and three invariance principles can be formulated: The invariance of the image amplitude with respect to (I) the slicing value $\delta\nu$, (II) the longitudinal relaxation time T_1, and (III) the initial conditions for the amplitude $A_\mu(0)$ and phase $\phi_\mu(0)$. To visualize the three invariance principles, RASER signals and corresponding Fourier transformed images are depicted in Fig. S12.

Fig. S12(A,B) shows invariance principle I. The absolute phased RASER image shape in (B) is invariant with respect to the slicing $\delta\nu$ even though the maxima of the three simulated RASER signals in (A) are shifted by a few 10 ms. For large $\delta\nu$ such as 0.4 Hz close to the linewidth w = 0.45 Hz the RASER image is characterized by small ripples, indicating insufficient digitization. However, as long as $\delta\nu \ll$ w, the amplitude of the RASER image is invariant with respect to the slicing $\delta\nu$. In Fig. S12(B) this is the case for $\delta\nu$ < 0.35 Hz and depicted for $\delta\nu$ = 0.25 Hz and 0.16 Hz. For $\delta\nu \to 0$, a continuous model for RASER MRI

is obtained. This model is characterized by an infinite number of slices where the sums in Eqs.(S5-S8) are replaced by integrals.
In Fig. S12(C, D), the invariance principle II is shown based on the three different T_1 relaxation times (T_1 = 10 s, 20 s, and 50 s). The signals in (C) are nearly identical and only shifted in time by < 20 ms. The corresponding images in (D) are almost identical. For T_1 = 10 s, the image differs by a few percent as compared to $T_1 \geq 20$ s. The reason for this difference is not the shift in time, but the decay of the RASER signal amplitude during the few seconds of the RASER activity. For $T_1 > 50$ s, the differences in the image amplitude becomes negligibly small.
The last invariance principle III is demonstrated in Fig. S12(E,F). Three identical images are obtained for three different initial conditions A_μ (0) and ϕ_μ (0). The case of a small flip angle is simulated as $A_\mu(0) = 5{\cdot}10^9$ and $\phi_\mu(0) = \pi/2$ (green). Additionally, two random initial conditions are assumed with $A_\mu(0) = 10^{10}{\cdot}$Random[] and $\phi_\mu(0) = 2\pi{\cdot}$Random[] (red and blue dots). These two random initial conditions correspond to nuclear spin noise, which can be composed of quantum fluctuations of nuclear magnetization, Nyquist noise of the coil, and Nyquist noise of the preamp (*26*). For RASER MRI it makes no difference which effect is responsible for the nuclear spin noise. All three initial conditions are displayed in the two insets in Fig. S12(E). The maximum amplitudes of the three RASER bursts are shifted in time by several 100 ms, but their envelopes are identical. The three corresponding Fourier transformed images in (F) cannot be distinguished in the absolute mode. However, they are shifted by a global phase, which can be seen in their phased FT spectra (not shown in (F)).

7. Conclusion & Outlook

In conclusion we have presented the theory of RASER MRI, which in the imaging domain Δ can be approximated by a network of $N = \Delta/\delta\nu$ equidistant nonlinear coupled slices (Eqs.(S5-S8)). Various nonlinear effects, such as high sensitivity to local variations in the input profile $\rho_d(\nu)$, amplitude deformations and edge artefacts, are predicted by the theory and indeed have been observed in various experiments. Interestingly, the mathematical form of Eqs.(S5-S8) is closely related to many descriptions of nonlinear and collective phenomena in other fields of science. Especially self-organized processes, the topic of synergetics uses order parameters and adiabatic elimination of fast variables to derive the LASER equations. Looking at the multimode case, H. Haken has shown a close relationship between the LASER assuming a continuous number of modes with the Ginzburg-Landau theory of superconductivity (*15*). A similar correspondence between uniformly distributed oscillators with non-local coupling with the Ginzburg-Landau theory of superconductivity is shown in the article by Y. Kuramoto and D. Battogktokh (*35*). In fact, the Kuramoto model of synchronized oscillators, which is equivalent to Eq.(S7), is a subset of our model of RASER MRI. As outlined in the book from S. Strogatz (*14*) and in many articles (*35, 36, 53*) phase synchronism in non-linear coupled oscillators occur in many different fields in physics and biology. Examples are biological rhythms, synchronized action of fireflies and tree frogs, electrically coupled Josephson arrays (*14*), mechanically coupled metronomes (chimera states) (*54*), spin torque nano-oscillators (*48*), synchronized spin-valve oscillators (*55*), and the dynamics of neural oscillator networks (*49*).

Furthermore Haken's equations for one single LASER mode without applying the enslaving principle are, apart from a variable transformation, identical to the pioneering Lorenz equations (*14, 56*), the latter of which describes chaos in a three dimensional space (*15*). In a similar manner it has been shown that chaos arises for the case of two enslaved RASER modes (*16*), which involves the evolution of four independent parameters d_1, α_1, d_2, and α_2. At present the exact analysis for why and how chaos arises in N coupled RASER modes is unknown.

Due to the strong links of Eqs.(S5-S8) to many different fields we believe that the presented theory of RASER MRI may be a base for a deeper understanding of self-organizing processes based on both adiabatic elimination of fast variables and on synchronism.

REFERENCES AND NOTES

1. M. G. Richards, B. P. Cowan, M. F. Secca, K. Machin, The ^{3}He nuclear Zeeman maser. *J. Phys. B. At. Mol. Opt.* **21**, 665–681 (1988).

2. T. E. Chupp, R. J. Hoare, R. L. Walsworth, B. Wu, Spin-exchange-pumped ^{3}He and ^{129}Xe Zeeman masers. *Phys. Rev. Lett.* **72**, 2363–2366 (1994).

3. H. Gilles, Y. Monfort, J. Hamel, ^{3}He maser for earth magnetic field measurement. *Rev. Sci. Instrum.* **74**, 4515–4520 (2003).

4. D. J. Marion, G. Huber, P. Berthault, H. Desvaux, Observation of noise-triggered chaotic emissions in an NMR-maser. *Chem Phys. Chem.* **9**, 1395–1401 (2008).

5. P. Bösiger, E. Brun, D. Meier, Solid-state nuclear spin-flip maser pumped by dynamic nuclear polarization. *Phys. Rev. Lett.* **38**, 602–605 (1977).

6. A. G. Zhuravrev, V. L. Berdinskiĭ, A. L. Buchachenko, Generation of high-frequency current by the products of a photochemical reaction. *JETP Lett.* **28**, 140 (1978).

7. H. Y. Chen, Y. Lee, S. Bowen, C. Hilty, Spontaneous emission of NMR signals in hyperpolarized proton spin systems. *J. Magn. Reson.* **208**, 204–209 (2011).

8. E. M. M. Weber, D. Kurzbach, D. Abergel, A DNP-hyperpolarized solid-state water NMR MASER: Observation and qualitative analysis. *Phys. Chem. Chem. Phys.* **21**, 21278–21286 (2019).

9. M. A. Hope, S. Björgvinsdóttir, C. P. Grey, L. Emsley, A magic angle spinning activated ^{17}O DNP raser. *J. Phys. Chem. Lett.* **12**, 345–349 (2021).

10. S. Appelt, G. Wäckerle, M. Mehring, Deviation from Berry's adiabatic geometric phase in a ^{131}Xe nuclear gyroscope. *Phys. Rev. Lett.* **72**, 3921–3924 (1994).

11. T. W. Kornack, R. K. Ghosh, M. V. Romalis, Nuclear spin gyroscope based on an atomic comagnetometer. *Phys. Rev. Lett.* **95**, 230801 (2005).

12. S. Appelt, A. Kentner, S. Lehmkuhl, B. Blümich, From LASER physics to the para-hydrogen pumped RASER. *Prog. Nucl. Magn. Reson. Spectrosc.* **114–115**, 1–32 (2019).

13. V. V. Soshenko, S. V. Bolshedvorskii, O. Rubinas, V. N. Sorokin, A. N. Smolyaninov, V. V. Vorobyov, A. V. Akimov, Nuclear spin gyroscope based on the nitrogen vacancy center in diamond. *Phys. Rev. Lett.* **126**, 197702 (2021).

14. S. H. Strogatz, *Nonlinear Dynamics and Chaos: With Applications to Physics, Biology, Chemistry, and Engineering* (Avalon Publishing, 2014).

15. H. Haken, *Synergertics: An Introduction* (Springer-Verlag, 1983).

16. S. Appelt, S. Lehmkuhl, S. Fleischer, B. Joalland, N. M. Ariyasingha, E. Y. Chekmenev, T. Theis, SABRE and PHIP pumped RASER and the route to chaos. *J. Magn. Reson.* **322**, 106815 (2021).

17. C. R. Bowers, D. P. Weitekamp, Transformation of symmetrization order to nuclear-spin magnetization by chemical reaction and nuclear magnetic resonance. *Phys. Rev. Lett.* **57**, 2645–2648 (1986).

18. R. W. Adams, J. A. Aguilar, K. D. Atkinson, M. J. Cowley, P. I. P. Elliott, S. B. Duckett, G. G. R. Green, I. G. Khazal, J. López-Serrano, D. C. Williamson, Reversible interactions with para-hydrogen enhance NMR sensitivity by polarization transfer. *Science* **323**, 1708–1711 (2009).

19. M. Suefke, S. Lehmkuhl, A. Liebisch, B. Blumich, S. Appelt, Para-hydrogen raser delivers sub-millihertz resolution in nuclear magnetic resonance. *Nat. Phys.* **13**, 568–572 (2017).

20. A. N. Pravdivtsev, F. D. Sönnichsen, J. B. Hövener, Continuous radio amplification by stimulated emission of radiation using parahydrogen induced polarization (PHIP-RASER) at 14 Tesla. *Chem. Phys. Chem.* **21**, 667–672 (2020).

21. B. Joalland, N. M. Ariyasingha, S. Lehmkuhl, T. Theis, S. Appelt, E. Y. Chekmenev, Parahydrogen-induced radio amplification by stimulated emission of radiation. *Angew. Chem. Int. Ed.* **59**, 8654–8660 (2020).

22. P. T. Callaghan, *Principles of Nuclear Magnetic Resonance Microscopy* (Clarendon Press, 1993).

23. P. C. Lauterbur, P. Mansfield, *The Nobel Prize in Physiology or Medicine* (2003); www.nobelprize.org/prizes/medicine/2003/summary/.

24. A. Vlassenbroek, J. Jeener, P. Broekaert, Radiation damping in high resolution liquid NMR: A simulation study. *J. Chem. Phys.* **103**, 5886–5897 (1995).

25. Y. Y. Lin, N. Lisitza, S. Ahn, W. S. Warren, Resurrection of crushed magnetization and chaotic dynamics in solution NMR spectroscopy. *Science* **290**, 118–121 (2000).

26. M. T. Pöschko, V. V. Rodin, J. Schlagnitweit, N. Müller, H. Desvaux, Nonlinear detection of secondary isotopic chemical shifts in NMR through spin noise. *Nat. Commun.* **8**, 13914 (2017).

27. N. Müller, A. Jerschow, Nuclear spin noise imaging. *Proc. Natl. Acad. Sci. U.S.A.* **103**, 6790–6792 (2006).

28. M. Suefke, A. Liebisch, B. Blümich, S. Appelt, External high-quality-factor resonator tunes up nuclear magnetic resonance. *Nat. Phys.* **11**, 767–771 (2015).

29. M. P. Augustine, S. D. Bush, E. L. Hahn, Noise triggering of radiation damping from the inverted state. *Chem. Phys. Lett.* **322**, 111–118 (2000).

30. A. Jurkiewicz, Properties and edition of NMR spontaneous maser emission spectra. *Appl. Magn. Reson.* **50**, 709–724 (2019).

31. X. A. Mao, C. H. Ye, Understanding radiation damping in a simple way. *Concept. Magn. Reson.* **9**, 173–187 (1997).

32. M. P. Augustine, Transient properties of radiation damping. *Prog. Nucl. Magn. Reson. Spectrosc.* **40**, 111–150 (2002).

33. V. V. Krishnan, N. Murali, Radiation damping in modern NMR experiments: Progress and challenges. *Prog. Nucl. Magn. Reson. Spectrosc.* **68**, 41–57 (2013).

34. S. H. Strogatz, From Kuramoto to Crawford: Exploring the onset of synchronization in populations of coupled oscillators. *Physica D* **143**, 1–20 (2000).

35. Y. Kuramoto, D. Battogtokh, Coexistence of coherence and incoherence in nonlocally coupled phase oscillators. *Nonlinear Phenom. Complex Syst.* **5**, 380 (2002).

36. Y. Kuramoto, H. Nakao, On the concept of dynamical reduction: The case of coupled oscillators. *Philos. Trans. Royal Soc. A* **377**, 20190041 (2019).

37. X. A. Mao, C. H. Ye, Line shapes of strongly radiation-damped nuclear magnetic resonance signals. *J. Chem. Phys.* **99**, 7455–7462 (1993).

38. X. A. Mao, J. X. Guo, C. H. Ye, Nuclear-magnetic-resonance line-shape theory in the presence of radiation damping. *Phys. Rev. B* **49**, 15702–15711 (1994).

39. D. J. Y. Marion, P. Berthault, H. Desvaux, Spectral and temporal features of multiple spontaneous NMR-maser emissions. *Eur. Phys. J. D* **51**, 357–367 (2009).

40. V. Henner, H. Desvaux, T. Belozerova, D. J. Y. Marion, P. Kharebov, A. Klots, Collective effects due to dipolar fields as the origin of the extremely random behavior in hyperpolarized NMR maser: A theoretical and numerical study. *J. Chem. Phys.* **139**, 144111 (2013).

41. M. J. Cowley, R. W. Adams, K. D. Atkinson, M. C. R. Cockett, S. B. Duckett, G. G. R. Green, J. A. B. Lohman, R. Kerssebaum, D. Kilgour, R. E. Mewis, Iridium N-heterocyclic carbene complexes as efficient catalysts for magnetization transfer from para-hydrogen. *J. Am. Chem. Soc.* **133**, 6134–6137 (2011).

42. S.-C. Lee, K. Kim, J. Kim, S. Lee, J. H. Yi, S. W. Kim, K.-S. Ha, C. Cheong, One micrometer resolution NMR microscopy. *J. Magn. Reson.* **150**, 207–213 (2001).

43. L. Ciobanu, D. A. Seeber, C. H. Pennington, 3D MR microscopy with resolution 3.7 microm by 3.3 microm by 3.3 microm. *J. Magn. Reson.* **158**, 178–182 (2002).

44. A. J. Ilott, A. Jerschow, Super-resolution surface microscopy of conductors using magnetic resonance. *Sci. Rep.* **7**, 5425 (2017).

45. J. Schlagnitweit, S. W. Morgan, M. Nausner, N. Müller, H. Desvaux, Non-linear signal detection improvement by radiation damping in single-pulse NMR spectra. *Chem. Phys. Chem.* **13**, 482–487 (2012).

46. B. Joalland, T. Theis, S. Appelt, E. Y. Chekmenev, Background-free proton NMR spectroscopy with radiofrequency amplification by stimulated emission radiation. *Angew. Chem. Int. Ed.* **60**, 26298–26302 (2021).

47. J. De Wilde, D. Grainger, D. Price, C. Renaud, Magnetic resonance imaging safety issues including an analysis of recorded incidents within the UK. *Prog. Nucl. Magn. Reson. Spectrosc.* **51**, 37–48 (2007).

48. S. Kaka, M. R. Pufall, W. H. Rippard, T. J. Silva, S. E. Russek, J. A. Katine, Mutual phase-locking of microwave spin torque nano-oscillators. *Nature* **437**, 389–392 (2005).

49. C. Bick, M. Goodfellow, C. R. Laing, E. A. Martens, Understanding the dynamics of biological and neural oscillator networks through exact mean-field reductions: A review. *J. Math. Neurosci.* **10**, 9 (2020).

50. J. T. Vaughan, M. Garwood, C. M. Collins, W. Liu, L. D. Barre, G. Adriany, P. Andersen, H. Merkle, R. Goebel, M. B. Smith, K. Ugurbil, 7T vs. 4T: RF power, homogeneity, and signal-to-noise comparison in head images. *Magn. Reson. Med.* **46**, 24–30 (2001).

51. F. Engelke, Virtual photons in magnetic resonance. *Concepts Magn. Reson. Part A* **36A**, 266–339 (2010).

52. X. A. Mao, Calculation of the energy transferred by radiation damping from nuclear spin system to receiver coil in NMR. *Chem. Phys. Lett.* **756**, 137853 (2020).

53. S. Nadis, All together now. *Nature* **421**, 780–782 (2003).

54. P. Ebrahimzadeh, M. Schiek, P. Jaros, T. Kapitaniak, S. van Waasen, Y. Maistrenko, Minimal chimera states in phase-lag coupled mechanical oscillators. *Eur. Phys. J. Spec. Top.* **229**, 2205–2214 (2020).

55. A. Ruotolo, V. Cros, B. Georges, A. Dussaux, J. Grollier, C. Deranlot, R. Guillemet, K. Bouzehouane, S. Fusil, A. Fert, Phase-locking of magnetic vortices mediated by antivortices. *Nat. Nanotechnol.* **4**, 528–532 (2009).

56. E. N. Lorenz, Deterministic nonperiodic flow. *J. Atmos. Sci.* **20**, 130–141 (1963).
